# Risk Factors for Recurrent Urinary Tract Infections Among Women in a Large Integrated Health Care Organization in the United States

**DOI:** 10.1093/infdis/jiae331

**Published:** 2024-06-28

**Authors:** Bradley K Ackerson, Sara Y Tartof, Lie H Chen, Richard Contreras, Iris Anne C Reyes, Jennifer H Ku, Michele Pellegrini, Johannes E Schmidt, Katia J Bruxvoort

**Affiliations:** Department of Research and Evaluation, Kaiser Permanente Southern California, Pasadena, CA, USA; Department of Research and Evaluation, Kaiser Permanente Southern California, Pasadena, CA, USA; Department of Research and Evaluation, Kaiser Permanente Southern California, Pasadena, CA, USA; Department of Research and Evaluation, Kaiser Permanente Southern California, Pasadena, CA, USA; Department of Research and Evaluation, Kaiser Permanente Southern California, Pasadena, CA, USA; Department of Research and Evaluation, Kaiser Permanente Southern California, Pasadena, CA, USA; GlaxoSmithKline, Siena, Italy; GlaxoSmithKline, Siena, Italy; Department of Epidemiology, University of Alabama at Birmingham, Birmingham, AL, USA

**Keywords:** recurrence, risk factors, urinary tract infection, UTI, women

## Abstract

**Background:**

Urinary tract infections (UTIs) occur commonly and often recur. However, recent data on the epidemiology of recurrent UTI (rUTI) are scarce.

**Methods:**

Between 1 January 2016 and 31 December 2020, index uncomplicated UTIs (uUTIs) from office, emergency department, hospital, and virtual care settings were identified from the electronic health records of women at Kaiser Permanente Southern California. We defined rUTI as ≥3 UTIs within 365 days or ≥2 UTIs within 180 days. We determined the proportion of women with cystitis index uUTI who had rUTI, and we examined factors associated with rUTIs using modified multivariable Poisson regression.

**Results:**

Among 374 171 women with cystitis index uUTI, 54 318 (14.5%) had rUTI. A higher proportion of women with rUTI vs those without rUTI were aged 18 to 27 or ≥78 years at index uUTI (19.7% vs 18.7% and 9.0% vs 6.0%, respectively), were immunocompromised, or had a positive urine culture result at index uUTI. In multivariable analyses, characteristics associated with rUTI included younger or older age (48–57 vs 18–27 years: adjusted risk ratio [aRR], 0.83 [95% CI, .80–.85]; ≥78 vs 18–27 years: aRR, 1.07 [95% CI, 1.03–1.11]), Charlson Comorbidity Index (≥3 vs 0: aRR, 1.12 [95% CI, 1.08–1.17]), and diabetes mellitus (aRR, 1.07 [95% CI, 1.04–1.10]). More frequent prior-year outpatient and emergency department encounters, oral antibiotic and oral contraceptive prescriptions, positive culture result at index uUTI, and antibiotic-resistant organisms were also associated with increased risk of rUTI.

**Conclusions:**

The high risk of rUTI among women with cystitis is concerning, especially given previous reports of increasing UTI incidence. Current assessment of the epidemiology of rUTI may guide the development of preventive interventions against UTI.

## Teaser

Recurrent urinary tract infections occurred commonly in a large cohort of women and were associated with ages 18 to 27 or ≥78 years, comorbidities, history of frequent health care utilization and antibiotic prescriptions, and antibiotic-resistant urine pathogens.

Urinary tract infections (UTIs) are among the most common bacterial infections [1], with an estimated annual incidence of >150 million cases worldwide [2]. In the United States, UTIs result in approximately 10.5 million office visits as well as 3 million emergency department (ED) encounters and 400 000 hospitalizations annually with an estimated annual cost >$4.8 billion [3–5]. UTIs primarily affect women, followed by male infants and older men [6]. Based on epidemiologic data from a study in the United States, about 1 in 3 women have at least 1 UTI diagnosed by a clinician that requires antibiotic medication, and the lifetime risk of UTIs among women is >60% [7].

Clinically, UTIs are categorized as uncomplicated or complicated. Uncomplicated UTIs (uUTIs) can be differentiated into lower (cystitis) and upper (pyelonephritis). uUTIs typically affect individuals who are otherwise healthy [8]. Approximately 75% of uUTIs are caused by extraintestinal pathogenic *Escherichia coli*, followed by *Klebsiella pneumoniae* and other pathogens [5]. Complicated UTIs are associated with factors compromising the urinary tract or host defense, such as urinary obstruction, immunosuppression, renal failure/transplantation, and indwelling catheterization [5]. In the United States, 70% to 80% of complicated UTIs are attributable to indwelling catheters, accounting for 1 million cases per year [9].

Antibiotics, which are the mainstay of UTI treatment, are often prescribed for UTIs in the absence of urine culture and antibiotic susceptibility testing [10]. However, antibiotic resistance by pathogens causing UTIs, particularly *E coli*, has been increasing, resulting in higher risk of treatment failure, patient morbidity, health care costs, and use of broad-spectrum antibiotics [5]. In addition, antibiotic resistance can limit oral therapeutic options for outpatient treatment of uUTIs, leading to greater hospital admissions, further escalating costs [11]. Furthermore, because of alterations in the gut microbiome, antibiotic administration may increase the risk of recurrent UTIs (rUTIs) [12].

UTIs have a propensity to recur, often more than once. UTI recurrences have been defined several ways: as a second or third UTI within 6 months of an index UTI, as a second or third UTI within 12 months of an index UTI [13, 14], or as a second UTI within 6 months or a third UTI within 12 months of an index UTI [15–17]. One study found that 24% of college women with a first UTI experienced a recurrence within 6 months [18], while another study reported that women with a history of ≥2 UTIs had 2 to 5 times the risk of recurrence within 1 year as compared with women with ≤1 UTI [19]. A nationwide US study of self-reported UTIs revealed that most UTIs occurred among women with a history of ≥2 UTI episodes [7]. However, despite the high frequency of UTIs and recurrences and their great burden on the health care system and patients, recent data on the epidemiology of rUTIs in the United States are limited. While several earlier studies examined risk factors for rUTIs, most evaluated a limited number of participants [13–15, 20], and many focused on specific populations, such as children [21], young women [13, 15, 18, 19], and older adults [16]. Furthermore, outpatient UTI rates have been rising, especially in virtual care settings, a trend that has likely accelerated during the COVID-19 pandemic [22]. Hence, a current assessment of the epidemiology of rUTIs may help guide the development and use of preventive interventions against UTI, including vaccines currently under development [23]. Therefore, we evaluated the epidemiology of rUTIs after uUTI among women in a large diverse population.

## METHODS

### Study Setting

Kaiser Permanente Southern California (KPSC) is an integrated health care system that provides comprehensive prepaid health services for >4.8 million members at 15 hospitals and 233 medical offices. The demographic makeup of the KPSC membership mirrors the racially and sociodemographically diverse southern California population and the California census population [24]. Comprehensive electronic health records used for this study contain information on demographics, services, diagnoses, procedures, laboratory results, pharmacy records, and immunizations from the outpatient (including virtual), ED, and hospital settings. Each KPSC health plan member has a unique medical record number used as an identifier to retrieve and link all variables from different databases. As KPSC is a prepaid health care system, there is strong motivation for members to use services internally. Furthermore, reimbursement by KPSC for outside care requires that claims be submitted with documentation of the care provided. Thus, capture of care delivered to KPSC members by electronic data is very comprehensive.

### Study Population

The study population comprised adults ≥18 years of age who sought outpatient care for UTI at KPSC between 1 January 2016 and 31 December 2020. We identified occurrences of UTI from office, ED, hospital, and virtual care (phone, video and internet) encounters. UTI was defined as 1 of the following: (1) occurrence of a UTI diagnosis code with a prespecified antibiotic prescription order (aminoglycoside, carbapenem, cephalosporin, fluoroquinolone, fosfomycin, nitrofurantoin, penicillin, trimethoprim-sulfamethoxazole, or other) ±3 days of a UTI diagnosis code date, (2) a positive urine culture result with a prespecified antibiotic prescription order ±3 days of culture date, or (3) a positive urine culture result with a UTI diagnosis code ±7 days of culture date (supplementary material). Positive urine culture results were defined according to KPSC laboratory guidelines as isolation of ≥1000 colony-forming units (CFU)/mL for normally sterile samples or ≥10 000 CFU/mL for clean-catch specimens. Since laboratory results provided by the KPSC laboratory to clinicians are based on these thresholds, we used these rather than higher thresholds (eg, ≥100 000 CFU/mL) to reflect real-world practice [25].

UTI occurrences were then categorized as uUTI (an infection in the absence of structural or functional abnormalities) or complicated UTI (infection in the presence of structural or functional abnormalities). The first uUTI was considered the index uUTI. Index uUTI type was further defined as pyelonephritis, cystitis, or other (unable to define due to UTI determined by positive culture with prespecified antibiotic prescription without a UTI diagnosis code; Supplementary Material, step 1). Since women accounted for >96% of participants with uUTIs with subsequent rUTIs and nearly 90% of rUTIs occurred among participants whose initial uUTI was cystitis, we focused the primary analysis on rUTIs among women with cystitis index uUTI.

Participants were required to have continuous KPSC membership for ≥1 year (allowing a 31-day gap) prior to the index uUTI to allow for ascertainment of baseline characteristics. Members with UTI within 1 year prior to the index uUTI were excluded from analysis. Participants were followed from the date of the index uUTI for occurrence of UTI recurrences (uUTI or complicated UTI).

rUTIs were defined as ≥3 UTI events (index uUTI plus ≥2 UTIs) within 365 days or ≥2 UTI events (eg, index uUTI plus ≥1 UTI) within 180 days [15–17, 26]. To define distinct UTI episodes as opposed to multiple records for the same infection, UTI occurrences within <31 days were grouped, with a gap ≥31 days required to define separate UTI episodes, as previously described [22].

### Covariates

We examined the baseline characteristics of participants in the year prior to the index uUTI, as stratified between those with and those without rUTI: age group (18–27, 28–37, 38–47, 48–57, 58–67, ≥68 years), race and ethnicity (White, Black, Hispanic, Asian/Pacific Islander, other/unknown), most recent body mass index (BMI; <18.5, 18.5–24.9, 25.0–29.9, 30.0–39.9, ≥40.0), and diabetes mellitus (defined by Charlson diagnosis codes for diabetes with and without complications), as well as smoking status (never, ever), immunocompromised status as previously described [27], Charlson Comorbidity Index (0, 1–2, ≥3) [28], renal disease, dementia, pregnancy, oral contraceptive prescriptions, number of health care encounters (outpatient, ED, and hospitalization), and number and class of antibiotic prescriptions (0, 1–2, ≥3). Other variables were neighborhood-level income, receipt of Medicaid insurance (yes/no), whether the UTI was confirmed with a positive urine culture result (vs no culture or a negative result), year of index uUTI, and the UTI pathogens and susceptibility for those identified with positive cultures (susceptible or nonsusceptible to 1–2 or ≥3 antibiotic classes: aminoglycosides, carbapenems, cephalosporins, fluoroquinolones, fosfomycin, nitrofurantoin, penicillins, trimethoprim-sulfamethoxazole, others).

### Statistical Analysis

Differences in sociodemographics, baseline characteristics, treatments, and other covariates were compared by rUTI status. Categorical data were summarized as frequencies and percentages and compared by χ^2^ test or Fisher exact test. Continuous variables were described as mean with SD or median with IQR and were compared by Student *t* test or the nonparametric Wilcoxon rank sum test (Mann-Whitney *U* test). The proportion of individuals with rUTI was calculated as the number of rUTI events divided by the number of participants with an index uUTI and was stratified by characteristics such as age, sex, race and ethnicity, history of UTI, and diabetes.

To examine factors associated with rUTI, unadjusted and adjusted risk ratios (aRRs) and 95% CIs were estimated through modified Poisson regression models with robust variance estimation. The multivariable model was developed via the LASSO method (least absolute shrinkage and selection operator). Variables in the final model were age, race and ethnicity, BMI, neighborhood-level income, Medicaid, Charlson Comorbidity Index, diabetes, dementia, pregnancy, oral contraceptives, health care utilization history in the year prior to the index date (numbers of outpatient, ED, and inpatient encounters), immunocompromised status at index, antibiotic prescription numbers in the year prior to the index date and class (cephalosporin, fluoroquinolone, nitrofurantoin), year of index uUTI, and positive urine culture result. We conducted similar analyses stratified by race and ethnicity, as well as in the subset of participants identified with positive culture results with additional variables that included urinary pathogens and nonsusceptibility to antibiotics. All analyses were conducted with SAS version 9.4 (SAS Institute).

This study was reviewed and approved by the KPSC Institutional Review Board.

## RESULTS

**Figure 1.**
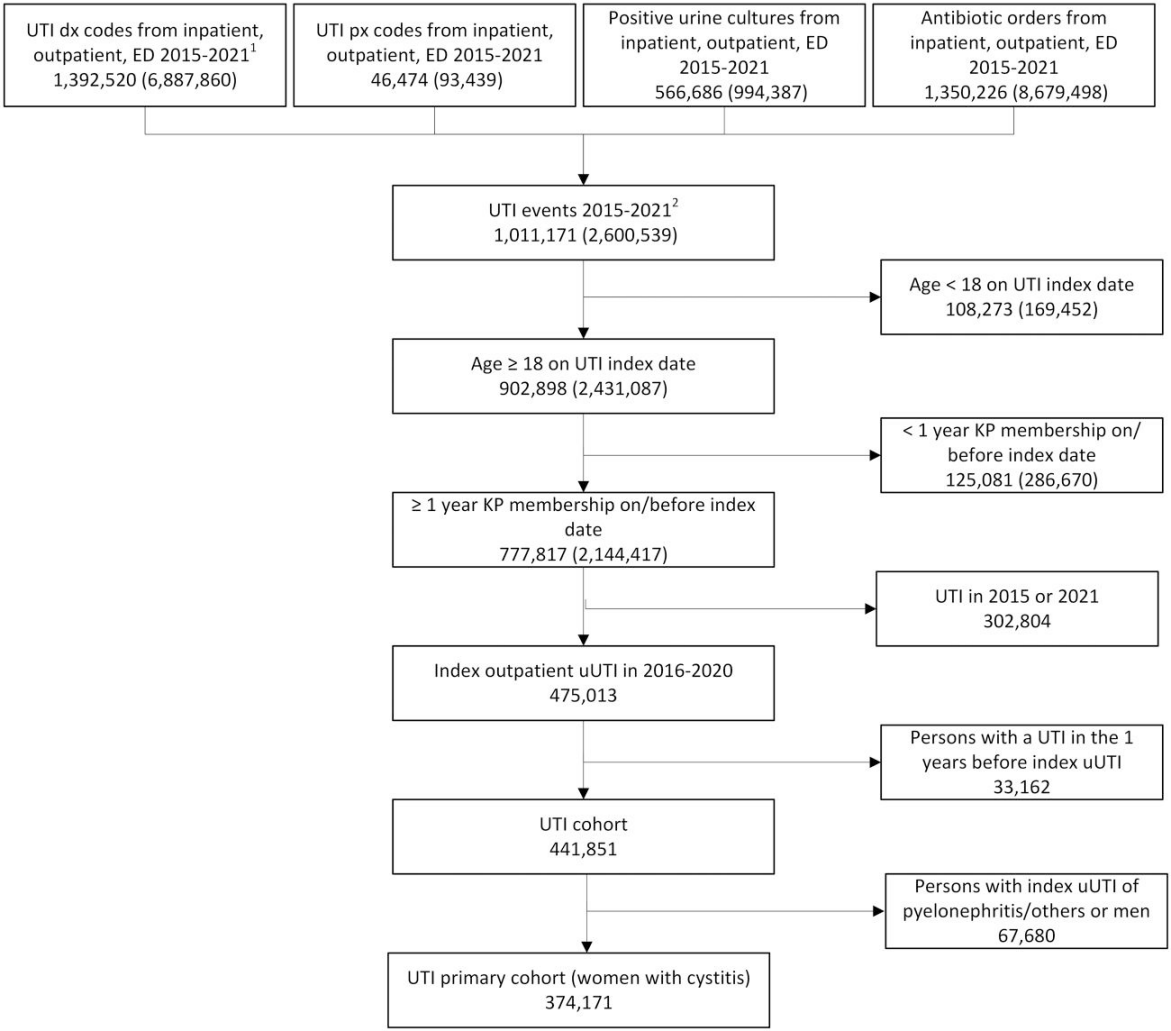
Flowchart for the study cohort. ^1^ Including e-visits and telephone appointment visits. ^2^ Number of unique persons (number of UTI events in parentheses). ^3^ The index date was the date of the earliest record for the first uUTI per person. dx, diagnosis; ED, emergency department; KP, Kaiser Permanente Southern California; px, procedure; UTI, urinary tract infection; uUTI, uncomplicated urinary tract infection.

During 2015 to 2021, there were 2 600 539 UTI events, of which 2 144 427 occurred among 777 871 KPSC members ≥18 years of age who had ≥1 year of prior KPSC membership (Figure 1). Among these, 475 013 members had an outpatient index uUTI during 2016 to 2020, of whom 33 162 were excluded due to having a UTI in the prior year, leaving 441 851 members in the overall uUTI cohort. The final cohort of women with cystitis as their index uUTI comprised 374 171 members.

Among the overall uUTI cohort, 92.2% (n = 407 479) were female, and 90.3% (n = 399 003) had cystitis as their index uUTI (Supplementary Table 1).

Among the final analytic cohort of 374 171 female participants with cystitis at the index uUTI, 54 318 (14.5%) had rUTI by our primary definition (≥3 UTIs [index uUTI plus ≥2 UTIs] within 365 days or ≥2 UTIs [index uUTI plus ≥1 UTI] within 180 days; Table 1), as opposed to 5.7% by a more restrictive definition (≥3 UTIs [index uUTI plus ≥2 UTIs] within 365 days) and 23.1% by a more permissive definition [17] (≥2 UTIs [index uUTI plus ≥1 UTI] within 365 days). The median age was 46.9 years, 45% were Hispanic and 34% were non-Hispanic White, and 13.1% had diabetes. A higher proportion of women with rUTI vs those without were 18 to 27 or ≥78 years of age at the index uUTI (19.7% vs 18.69% and 9.0% vs 6.0%, respectively) or were immunocompromised or had a positive urine culture result at the index uUTI. In the year prior to the index uUTI, those with rUTI vs those without rUTI had lower BMI and diabetes and greater health care utilization, received oral contraceptives or antibiotic prescriptions, and were more likely to have a Charlson Comorbidity Index >0 (38.5% vs 32.9% with ≥1; Table 1).

**Table 1.** Baseline Characteristics of Women With Cystitis as the Index uUTI by rUTI, Kaiser Permanente Southern California, 2016–2021

	rUTI (n = 54 318)		No rUTI (n = 319 853)		Total (n = 374 171)		
	No.	%	No.	%	No.	%	*P* Value
Age group, y^a^							<.001
18–27	10 693	19.7	59 784	18.7	70 477	18.8	
28–37	9316	17.2	58 763	18.8	68 079	18.2	
38–47	7936	14.6	53 379	16.7	61 315	16.4	
48–57	7746	14.3	52 362	16.4	60 108	16.1	
58–67	7621	14.0	45 407	14.2	53 028	14.2	
68–77	6106	11.2	30 927	9.7	37 033	9.9	
≥78	4900	9.0	19 231	6.0	24 131	6.5	
Age, y, median (IQR)	47.0 (30.0–64.0)		45.0 (31.0–61.0)		45.0 (31.0–61.0)		<.001
Race and ethnicity							<.001
White	20 373	37.5	107 045	33.5	127 418	34.1	
Black	3992	7.3	26 996	8.4	30 988	8.3	
Hispanic	23 650	43.5	144 454	45.2	168 104	44.9	
Asian/Pacific Islander	4636	8.5	31 343	9.8	35 979	9.6	
Other/unknown	1667	3.1	10 015	3.1	11 682	3.1	
Body mass index^b^							<.001
<18.5	1030	1.9	5215	1.6	6245	1.7	
18.5–24.9	16 785	30.9	89 491	28.0	106 276	28.4	
25.0–29.9	14 821	27.3	86 368	27.0	101 189	27.0	
30.0–39.9	14 050	25.9	84 273	26.3	98 323	26.3	
≥40.0	3410	6.3	20 882	6.5	24 292	6.5	
Unknown	4222	7.8	33 624	10.5	37 846	10.1	
Smoking status^b^							<.001
Never	42 145	77.6	251 420	78.6	293 565	78.5	
Ever	11 127	20.5	61 615	19.3	72 742	19.4	
Unknown	1046	1.9	6818	2.1	7864	2.1	
Neighborhood-level income, $							<.001
<40 000	2088	3.8	12 580	3.9	14 668	3.9	
40 000–<60 000	9641	17.7	59 523	18.6	69 164	18.5	
60 000–<85 000	16 492	30.4	98 112	30.7	114 604	30.6	
≥85 000	25 783	47.5	148 048	46.3	173 831	46.5	
Unknown	314	0.6	1590	0.5	1904	0.5	
Medicaid	5953	11.0	30 967	9.7	36 920	9.9	<.001
Charlson Comorbidity Index score^b^							<.001
0	33 383	61.5	214 658	67.1	248 041	66.3	
1–2	16 635	30.6	89 020	27.8	105 655	28.2	
≥3	4300	7.9	16 175	5.1	20 475	5.5	
Diabetes^b^	8265	15.2	40 703	12.7	48 968	13.1	<.001
Renal disease^b^	4094	7.5	17 401	5.4	21 495	5.7	<.001
Dementia^b^	1243	2.3	4165	1.3	5408	1.4	<.001
Immunocompromised^a^	1490	2.7	6100	1.9	7590	2.0	<.001
Pregnancy^b^	525	1.0	4914	1.5	5439	1.5	<.001
Oral contraceptives^b^	4971	9.2	25 507	8.0	30 478	8.1	<.001
No. of outpatient visits^b^							<.001
0	1713	3.2	16 340	5.1	18 053	4.8	
1–4	13 919	25.6	101 274	31.7	115 193	30.8	
5–8	12 982	23.9	77 431	24.2	90 413	24.2	
9–15	12 871	23.7	67 323	21.0	80 194	21.4	
≥16	12 833	23.6	57 485	18.0	70 318	18.8	
No. of emergency department visits^b^							<.001
0	41 827	77.0	258 260	80.7	300 087	80.2	
1	8365	15.4	43 642	13.6	52 007	13.9	
≥2	4126	7.6	17 951	5.6	22 077	5.9	
No. of inpatient visits^b^							<.001
0	49 610	91.3	296 510	92.7	346 120	92.5	
1	3671	6.8	19 280	6.0	22 951	6.1	
≥2	1037	1.9	4063	1.3	5100	1.4	
No. of antibiotic prescriptions^b^							<.001
0	32 082	59.1	214 786	67.2	246 868	66.0	
1–2	16 961	31.2	86 062	26.9	103 023	27.5	
≥3	5275	9.7	19 005	5.9	24 280	6.5	
Antibiotic prescriptions class^b,c^							
Aminoglycoside	261	0.5	1396	0.4	1657	0.4	.153
Carbapenem	46	0.1	115	0.0	161	0.0	<.001
Cephalosporin	11 269	20.7	49 060	15.3	60 329	16.1	<.001
Fluoroquinolone	3928	7.2	13 506	4.2	17 434	4.7	<.001
Fosfomycin	18	0.0	24	0.0	42	0.0	<.001
Nitrofurantoin	2212	4.1	5805	1.8	8017	2.1	<.001
Penicillin	9979	18.4	52 140	16.3	62 119	16.6	<.001
Trimethoprim-sulfamethoxazole	2622	4.8	10 502	3.3	13 124	3.5	<.001
Others	556	1.0	2236	0.7	2792	0.7	<.001
Year of index uUTI							<.001
2016	11 993	22.1	69 145	21.6	81 138	21.7	
2017	11 246	20.7	67 458	21.1	78 704	21.0	
2018	11 320	20.8	65 966	20.6	77 286	20.7	
2019	10 012	18.4	61 259	19.2	71 271	19.0	
2020	9747	17.9	56 025	17.5	65 772	17.6	
Urine culture at index uUTI							<.001
None	31 466	57.9	187 044	58.5	218 510	58.4	
Negative	6080	11.2	48 672	15.2	54 752	14.6	
Positive	16 772	30.9	84 137	26.3	100 909	27.0	
No. with positive urine culture result^d^	16 727		83 854		100 581		
*E coli* isolated at index uUTI^e^	13 733	82.1	70 401	84.0	84 134	83.6	<.001
*Klebsiella* spp isolated at index uUTI^e^	1385	8.3	5323	6.3	6708	6.7	<.001
Nonsusceptibility to antibiotic classes							<.001
Susceptible	6984	41.8	36 921	44.0	43 905	43.7	
Nonsusceptible to 1–2 classes^f^	7444	44.5	36 949	44.1	44 393	44.1	
Nonsusceptible to ≥3 classes^f^	2299	13.7	9984	11.9	12 283	12.2	

Abbreviations: rUTI, recurrent urinary tract infection; uUTI, uncomplicated urinary tract infection.

^a^At index date (date of earliest record for the index uUTI).

^b^In the year prior to index date.

^c^Not mutually exclusive.

^d^Excludes cultures with unknown susceptibility.

^e^Other organisms isolated included *Proteus mirabilis* (4.29%), *Citrobacter* spp (1.59%), *Enterobacter spp* (1.51%), *Enterococcus* spp (0.92%), *Pseudomonas aeruginosa* (0.46%), coagulase-negative S*taphylococcus* spp (0.32%). More than 1 pathogen was isolated from 1.83% of positive culture results.

^f^Antibiotic classes were aminoglycosides, carbapenems, cephalosporins, fluroquinolones, fosfomycin, nitrofurantoin, penicillin, trimethoprim-sulfamethoxazole, others (aztreonam, colistin, daptomycin, linezolid, tigecycline, vancomycin).

**Figure 2.**
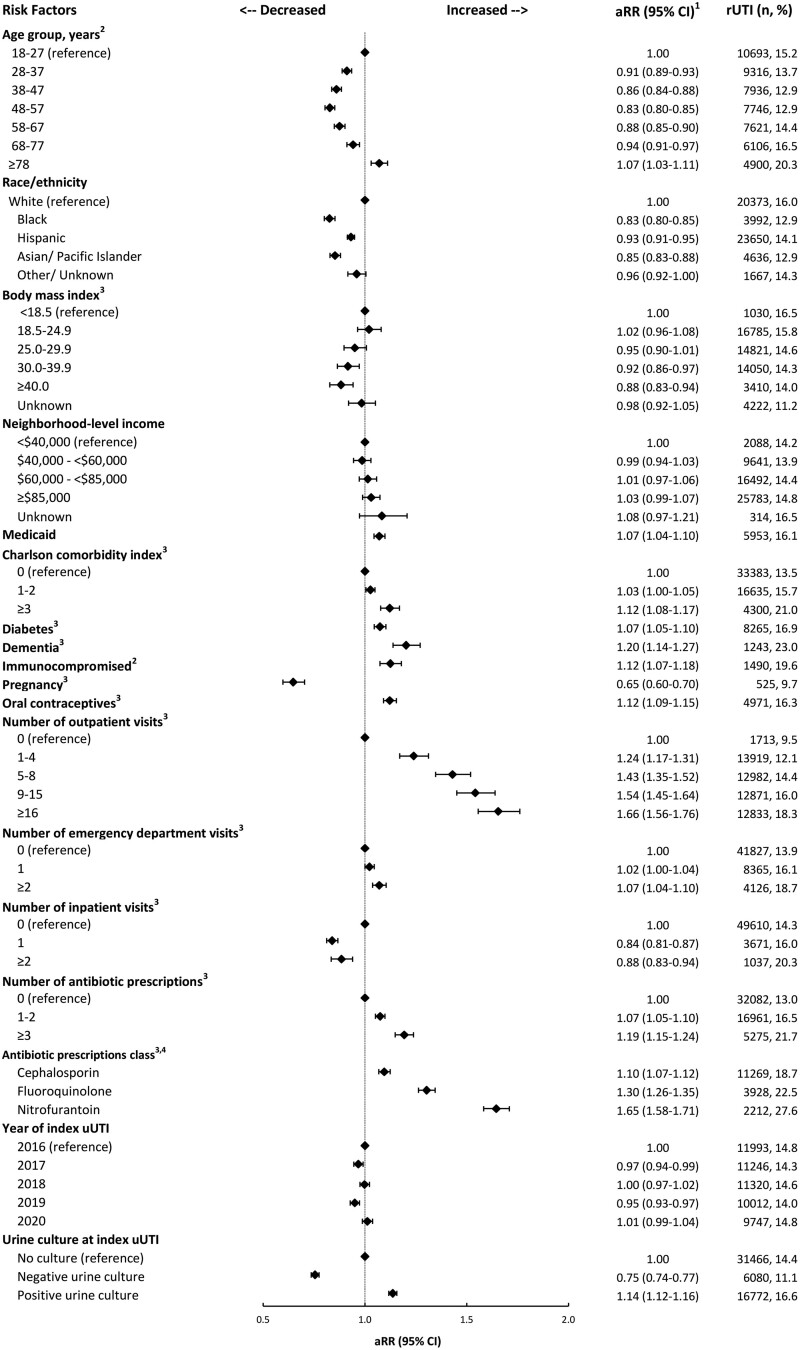
Factors associated with rUTI among women with cystitis index uUTI, Kaiser Permanente Southern California, 2016–2021. ^1^ aRR and 95% CI were estimated from modified Poisson regression models with a robust error variance, adjusted for all other variables in the figure. ^2^ At index date (date of the earliest record for the index uUTI). ^3^ In the year prior to the index date. ^4^ For each antibiotic class, the comparison group is not receiving an antibiotic of that class in the prior year. aRR, adjusted risk ratio; rUTI, recurrent urinary tract infection; uUTI, uncomplicated urinary tract infection.

In the adjusted analyses, characteristics associated with an increased risk of rUTI were as follows: younger or older age (eg, 48–57 vs 18–27 years: aRR, 0.83 [95% CI, .80–.85]; ≥78 vs 18–27 years: aRR, 1.07 [95% CI, 1.03–1.11]), White race and ethnicity (Black, Hispanic, or Asian/Pacific Islander vs White: aRR, 0.83 [95% CI, .80–.85], 0.93 [95% CI, .91–.95], and 0.85 [95% CI, .83–.88], respectively), receipt of Medicaid (aRR, 1.07 [95% CI, 1.04–1.10]), greater Charlson Comorbidity Index (≥3 vs 0: aRR, 1.12 [95% CI, 1.08–1.17]), diabetes (aRR, 1.07 [95% CI, 1.05–1.10]), dementia (aRR, 1.20 [95% CI, 1.14–1.18]), immunocompromised status (aRR, 1.12 [95% CI, 1.07–1.18]), more prior-year outpatient encounters (1–4, 5–8, 9–16, and >16 vs 0: aRR, 1.24, 1.43, 1.54, and 1.66, respectively), more prior-year ED encounters (≥2 vs 0: aRR, 1.07 [95% CI, 1.04–1.10]), receipt of more antibiotic prescriptions in the prior year (≥3 vs 0: aRR, 1.19 [95% CI, 1.15–1.24]), receipt of oral contraceptives in the prior year (aRR, 1.12 [95% CI, 1.09–1.15]), and a positive urine culture result at the index uUTI among those cultured (aRR, 1.14 [95% CI, 1.12–1.16]). In contrast, pregnancy was associated with a decreased risk of rUTI (aRR, 0.65 [95% CI, .60–.70]; Figure 2).

**Figure 3.**
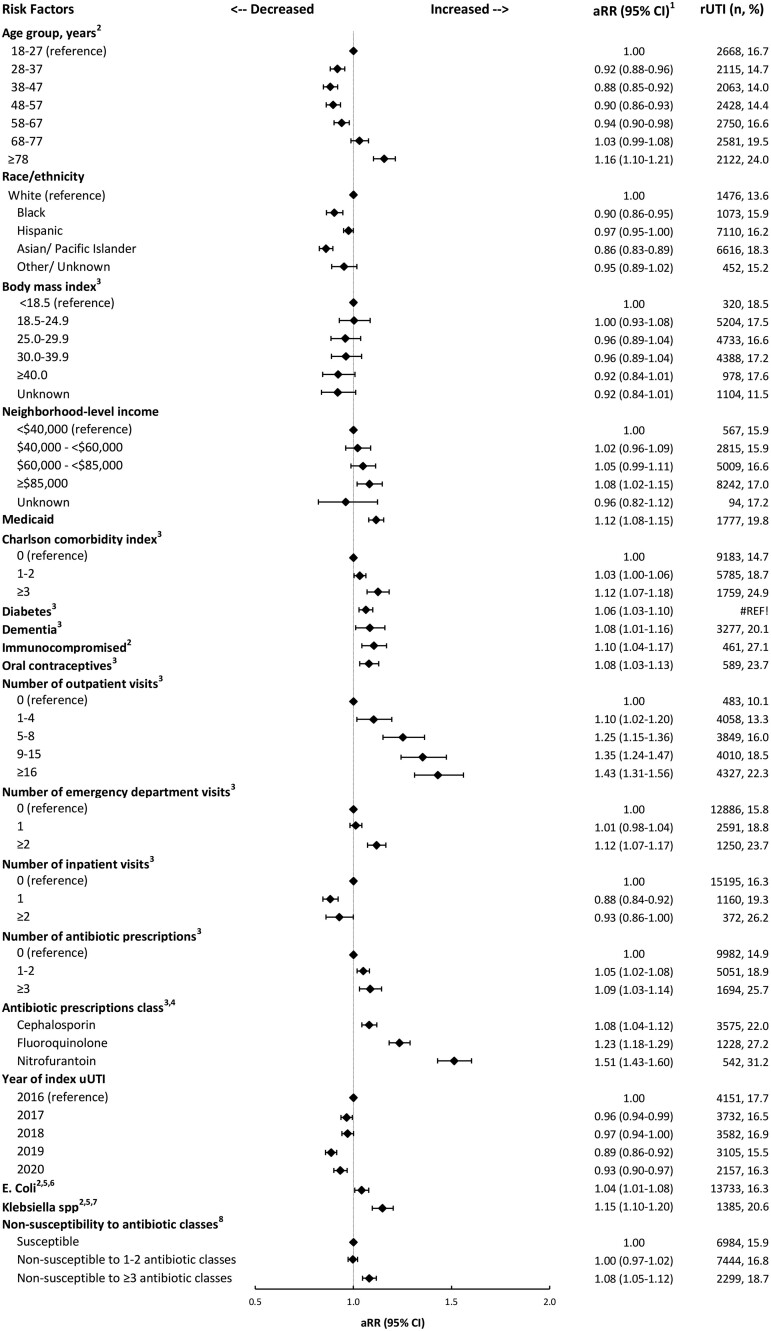
Factors associated with rUTI among women with cystitis index uUTI with positive culture results, Kaiser Permanente Southern California, 2016–2021. ^1^ aRR and 95% CI were estimated from modified Poisson regression models with a robust error variance, adjusted for all other variables in the figure. ^2^ At index date (date of the earliest record for the index uUTI). ^3^ In the year prior to the index date. ^4^ For each antibiotic class, the comparison group is not receiving an antibiotic of that class in the prior year. ^5^ Other organisms included *Proteus mirabilis* (4.29%), *Citrobacter* spp (1.59%), *Enterobacter* spp (1.51%), *Enterococcus* spp (0.92%), *Pseudomonas aeruginosa* (0.46%), and coagulase-negative *Staphylococcus* spp (0.32%). More than 1 organism was isolated from 1.83% of samples. ^6^*Escherichia coli* vs another organism isolated. ^7^*Klebsiella* spp vs another organism isolated. ^8^ Antibiotic classes were aminoglycosides, carbapenems, cephalosporins, fluroquinolones, fosfomycin, nitrofurantoin, penicillins, trimethoprim-sulfamethoxazole, others (aztreonam, colistin, daptomycin, linezolid, tigecycline, vancomycin). aRR, adjusted risk ratio; rUTI, recurrent urinary tract infection; uUTI, uncomplicated urinary tract infection.

In analyses among women with cystitis and a positive culture result for the index uUTI, *E coli* (isolated vs other organism isolated), *Klebsiella* spp (isolated vs other organism isolated), and nonsusceptibility to ≥3 classes of antibiotics were also associated with an increased risk of rUTI (aRR, 1.04 [95% CI, 1.01–1.08], 1.15 [95% CI, 1.10–1.20], and 1.08 [95% CI, 1.05–1.12], respectively; Figure 3).

The findings in the adjusted analysis among women with cystitis as their index uUTI stratified by race and ethnicity were similar across strata to those in the main analysis. Characteristics associated with a higher risk of rUTI in all race and ethnicity subgroups included age 18 to 27 years, dementia, positive urine culture result at the index uUTI, more outpatient encounters, receipt of ≥3 antibiotic prescriptions, and receipt of fluoroquinolones or nitrofurantoin in the year prior to the index uUTI. Furthermore, diabetes and receipt of oral contraceptives were associated with a greater risk of rUTI in all race and ethnicity subgroups except Black women, among whom the point estimate was >1 with wide confidence intervals overlapping 1. Similar patterns were seen for receipt of Medicaid and immunocompromise, which were associated with an increased risk of rUTI in all race and ethnicity groups except Asian/Pacific Islander women. As in the main analysis, pregnancy was associated with a decreased risk of rUTI among all race and ethnicity groups. Notably, age ≥78 years was not associated with a higher risk of rUTI among Black and Asian/Pacific Islander women as it was among other race and ethnicity groups and the main analysis (Supplementary Table 2)

The findings in the analyses among females with cystitis and a positive culture result for the index uUTI stratified by race and ethnicity paralleled those of the main analysis. Characteristics associated with a greater risk of rUTI in all race and ethnicity groups included younger age, receipt of Medicaid, nonsusceptibility to ≥3 classes of antibiotics, higher number of outpatient visits, and receipt of fluroquinolones or nitrofurantoin in the year prior to the index uUTI. Due to an insufficient sample size, characteristics associated with rUTI among the other/unknown cohort could not be analyzed in the fully adjusted model (Supplementary Table 3).

## DISCUSSION

This retrospective study spanning 5 years assessed risk factors associated with rUTI, an important source of morbidity [9] among women of all ages. This study describes one of the largest and most recent and diverse cohorts of women with rUTIs to date. We found that rUTI is common, occurring in nearly 15% of women following an incident uUTI, underscoring the high frequency of UTI recurrences and the substantial burden of UTIs in women. Prior studies have defined UTI recurrences in several ways [13–17]. Nevertheless, the rUTI rate that we observed (14.5%) is similar to that of ≥3 UTIs (index UTI plus ≥2 rUTIs) within 12 months of an index UTI observed in a smaller population (13%) [14]. However, it is lower than that of rUTI when defined as ≥2 UTIs (index UTI plus 1 rUTI) within 6 months of an index UTI in college-age women, who may be at greater risk of rUTI (26.6%) [13], and when defined more permissively as ≥2 UTIs (index UTI plus ≥1 rUTI) within 12 months of an index UTI (26%) [14].

Consistent with an earlier report, we found a bimodal age distribution for women with the greater risk of rUTIs among the youngest and oldest participants (18–27 and ≥78 years vs 28–67 years). The increased risk of rUTIs among younger women is thought to reflect behavioral risk factors, including sexual activity, and genetic risk factors [5, 16], including nonsecretor status and personal and familial history of UTI. The higher risk of rUTI among women who received oral contraceptive prescriptions in this study is similar to the greater risk of UTI associated with diaphragm use reported in prior studies [13], reflecting sexual activity as a likely risk factor for rUTI [29]. In contrast, hormonal changes [30] that increase colonization with uropathogens, as well as structural changes, are thought to have a greater role in increasing the risk of rUTIs among older women [16]. Similarly, in keeping with prior reports [18], in the main analysis, we found a higher incidence of rUTI among White participants vs participants of other races and ethnicities. Nonetheless, characteristics associated with greater risk of rUTI were mostly similar across subgroups in our analysis when stratified by race and ethnicity, although a higher risk of rUTI among older women in the main analysis was not seen among older Black and Asian/Pacific Islander women but was observed among older women of other races and ethnicities. However, sample size was limited for Black and Asian/Pacific Islander women in the oldest age groups, and observed differences in the risk of UTIs among races likely reflect unexamined confounders [31].

Several comorbidities were associated with an increased risk of rUTI: diabetes, immunocompromised status, dementia, and Charlson Comorbidity Index ≥1. Although diabetes is a known risk factor for UTI, likely through immunologic mechanisms [32], Raz found a nonsignificant higher risk of rUTI among women with diabetes, but those findings were likely limited by sample size [33]. While the impact of immunocompromised status on risk of rUTI has not been evaluated previously, it has been associated with a higher risk of UTI [34]. Likewise, a greater risk of rUTIs among persons with dementia was observed, mirroring a similar increased risk of UTIs in these individuals. We also noted a decreased risk of rUTI with pregnancy within 1 year of the index uUTI, possibly reflecting close monitoring and treatment of asymptomatic bacteriuria in pregnant women in this setting, which reduce the risk of UTI in pregnancy [35]. In addition, the numbers of antibiotic prescriptions and ED and outpatient visits within 1 year prior to an index uUTI were associated with a higher risk of rUTI, while the number of hospital admissions was not, possibly reflecting increased health care–seeking behavior or a greater number of conditions requiring health encounters in nonhospital settings.

Although our finding of a decreased risk of rUTI with higher BMI differs from studies that found the inverse association [36–38], other reports showed no association between BMI and risk of UTI in women or an inverse association [39, 40]. It can also be possible that risk factors for UTI may in part differ from those for rUTI, which have been less widely studied. Consistent with prior studies that reported a higher risk of symptomatic UTI following treatment of asymptomatic bacteriuria [41], we found that increased use of antibiotics (cephalosporins, fluoroquinolones, or nitrofuran), often used in outpatient settings, in the year prior to the index uUTI was associated with increased risk of rUTI in our study population, possibly due to antibiotic-associated changes in the gut and/or vaginal microbiota that appear to raise the risk of rUTI [12]. Similarly, multidrug resistance in isolates in women with cystitis whose urine was cultured was associated with an increased risk of rUTI, consistent with findings from previous studies [12, 42].

The strengths of this study include detailed clinical and health outcomes data extracted from a large patient sample drawn from a racially, socioeconomically, and age-diverse population receiving care at multiple medical centers over 5 years. In addition, this study focused on participants without UTI in the 12 months prior to the index uUTI, avoiding biases introduced by studying populations with recent UTI who are at higher risk of rUTI [14]. Limitations include the use of a threshold for positive culture that is lower than the threshold of ≥10^5^ CFU/mL used in some studies but is higher than that used in other prior studies [13, 15, 18]. Furthermore, the definition did not include clinical symptoms or dipstick or microscopic urinalysis, which are not consistently captured in the electronic health record. Instead, we created definitions using routinely collected electronic health record data that would be feasible to use across health systems [22]. Together, the limitations of our definitions improved the sensitivity of the diagnosis of UTI but may have resulted in inclusion of some members with asymptomatic bacteriuria. However, testing and treatment are typically prompted by clinical symptoms, and we required combinations of diagnosis codes, antibiotic prescriptions, and positive urine culture results, likely reducing the impact of this limitation.

In conclusion, we report the epidemiology of rUTI in a large, sociodemographically diverse population of women without a history of recent UTI, providing recent data that may be more generalizable than most earlier studies that focused on smaller cohorts of a limited age range or on cohorts with recent UTI or a history of rUTI that raised the risk of rUTI among their study populations. We found that rUTI following uUTI is common, particularly among younger and older women, those with comorbidities, and those with more antibiotic prescriptions in the year prior to the index uUTI or with an index uUTI caused by multidrug-resistant organisms. The rising incidence of UTI and worsening antibiotic resistance underscore the growing unmet medical and public health need to develop preventive measures directed against UTI, including vaccines [22, 23]. Current assessment of the epidemiology of rUTI provided by this report may help guide development and use of preventive therapies, including potential vaccines against common uropathogens.

## Supplementary Data

Supplementary materials are available at *The Journal of Infectious Diseases* online (http://jid.oxfordjournals.org/). Supplementary materials consist of data provided by the author that are published to benefit the reader. The posted materials are not copyedited. The contents of all supplementary data are the sole responsibility of the authors. Questions or messages regarding errors should be addressed to the author.

## Supplementary Material

jiae331_Supplementary_Data

## Notes

***Acknowledgments.*** We thank Pooja M. Patel, MPH, and Rossy F. Perez for their assistance in drafting the manuscript.

***Financial support*.** This work was supported by GlaxoSmithKline.
