# Supplementary material for: Risk Factors for Recurrent Urinary Tract Infections Among Women in a Large Integrated Health Care Organization in the United States
Source: J Infect Dis. 2024 Jun 28;230(5):e1101–11. doi: 10.1093/infdis/jiae331 (PMC11566237; doi:10.1093/infdis/jiae331)
Supplement: jiae331_Supplementary_Data [file jiae331_supplementary_data.docx]

**Supplementary Materials for:**

**Risk factors for recurrent urinary tract infections among women in a large integrated health care organization in the United States**

Ackerson B^1^, Tartof SY^1^, Chen LH^1^, Contreras R^1^, Reyes IA^1^, Ku J^1^, Pellegrini M^2^, Schmidt JE^2^, Bruxvoort KJ^3^

^1^Kaiser Permanente Southern California, Pasadena, CA, USA

^2^GlaxoSmithKline, Siena, Italy

^3^University of Alabama at Birmingham, Birmingham, AL, USA

**Table of Contents**

**Supplementary materials** Algorithm for identifying uncomplicated urinary tract infections......................2

**STable 1** Baseline characteristics of individuals in the UTI cohort, with and without rUTI, Kaiser Permanente Southern California, 2016-2021...............................................................................................8

**STable 2** Factors associated with rUTI among women with cystitis as index uUTI, by race/ethnicity.......10

**STable 3** Factors associated with rUTI among women with cystitis as index uUTI with positive urine culture, by race/ethnicity...........................................................................................................................12

**Supplementary Material**. Algorithm for identifying uncomplicated urinary tract infections.

Step 1 will identify all urinary tract infections (UTIs) from Kaiser Permanente Southern California (KPSC) electronic health records. Step 2 will categorize UTI as either uncomplicated (uUTI) or complicated UTI (cUTI).

**Step 1**

1. Identify all UTIs during 2015-2021 from outpatient^1^ and inpatient encounters as occurrence of any of the following criteria:

- [Step 1A] UTI diagnosis code with an antibiotic prescription order^2^ within ±3 days of diagnosis code date
- UTI diagnosis codes include any one of the following:
  - - Main UTI code:

ICD-10 N30.00, N30.01, N30.90, N30.91, N39.0, and N10

ICD-9 595.0, 595.9, 599.0, 590.10, 590.11

- - - Group A code.
    - Group B Infection code + Group C Structural abnormality/obstruction code or procedure, with the two codes within ≤30 days of each other.
- [Step 1B] Positive urine culture^3^ with an antibiotic prescription order within ±3 days of culture date
- [Step 1C] Positive urine culture with a UTI diagnosis code ±7 days of culture date
- UTI codes include ≥1 of the Main UTI codes, Group A codes, or Group B Infection + Group C Structural abnormality/obstruction codes.
  - - Group B + Group C codes need to be within ≤30 days of each other, with >1 of these codes ±7 days of culture date.

^1^Care setting will be defined as outpatient if all components of the UTI definition (diagnosis code, antibiotic order, or positive culture, as relevant) occur in the outpatient setting. Outpatient will include encounters categorized as outpatient, synchronous virtual (e-visit, video, telephone advise visit), emergency department, or other. Care setting will be defined as inpatient if any component of the UTI definition occurs in the inpatient setting, or if the emergency department care subtype includes an observation stay lasting >24 hours.

^2^List of antibiotics

| Class | Antibiotics |
| --- | --- |
| Aminoglycosides | amikacin, gentamicin, tobramycin, plazomicin |
| Carbapenems | doripenem, ertapenem, imipenem/cilastatin, meropenem |
| Cephalosporins | cefaclor, cefazolin, cefdinir, cefepime, cefixime, cefotaxime, cefpodoxime, ceftriaxone, ceftazidime, ceftazidime–avibactam, ceftolozane–tazobactam, cefuroxime, cephalexin, cefiderocol |
| Fluoroquinolones | ciprofloxacin, levofloxacin, moxifloxacin, ofloxacin |
| Fosfomycin | fosfomycin |
| Nitrofurantoin | nitrofurantoin |
| Penicillin | amoxicillin, amoxicillin-clavulanate, ampicillin, ampicillin-sulbactam, dicloxacillin, nafcillin, oxacillin, piperacillin-tazobactam |
| Trimethoprim-sulfamethoxazole | trimethoprim-sulfamethoxazole |
| Others | aztreonam, colistin, daptomycin, linezolid, tigecycline, vancomycin |

^3^Positive urine cultures will be defined as ≥1,000 colony-forming units (CFU)/mL for sterile samples and ≥10,000 CFU/mL for clean-catch samples, excluding cultures identified as contaminated by the KPSC laboratory and excluding *Candida spp*. For urine cultures missing CFU/mL or for those with multiple organisms identified, cultures will be considered positive if they have antibiotic susceptibility testing performed.

**Step 2**

1. Categorize as cUTI or uUTI

- For Step 1A
  1. Consider as cUTI if Main UTI code + either Group C Structural abnormality/obstruction code or procedure within ±30 days of Main UTI code and/or Group A code within ±30 days of Main UTI code.
  2. Consider as uUTI if Main UTI code + no Group C Structural abnormality/obstruction code or procedure within ±30 days of Main UTI code + no Group A code within ±30 days of Main UTI code.
  3. Consider as cUTI if Group A code.
  4. Consider as cUTI if Group B Infection code + Group C Structural abnormality/obstruction code or procedure, with the two codes within ≤30 days of each other.
- For Step 1B:
  1. Consider as cUTI if positive urine cultures with an antibiotic prescription order within ±3 days of culture date + a UTI diagnosis code ±7 days of culture date or antibiotic prescription order date, with the UTI diagnosis code being one of the following:
- Group A code.
- Group B Infection code + Group C Structural abnormality/obstruction code or procedure, with the two codes within ≤30 days of each other.
- Main UTI code + either Group C Structural abnormality/obstruction code or procedure within ±30 days of Main UTI code and/or Group A code within ±30 days of Main UTI code
  1. Consider as uUTI as if positive urine cultures with an antibiotic prescription order within ±3 days of culture date if UTI diagnosis code ±7 days of culture date or antibiotic prescription order date, with the UTI diagnosis code being one of the following:
     - - Main UTI code + no Group C Structural abnormality/obstruction code or procedure within ±30 days of Main UTI code + no Group A code within ±30 days of Main UTI code
  2. Consider as uUTI as if positive urine cultures with an antibiotic prescription order within ±3 days of culture date if no UTI diagnosis code ±7 days of culture date or antibiotic prescription order date
- For Step 1C:
  1. Consider as cUTI if positive urine culture with a UTI diagnosis code ±7 days of culture date, with the UTI diagnosis code being one of the following:
- Group A code.
- Group B Infection code + Group C Structural abnormality/obstruction code or procedure, with the two codes within ≤30 days of each other.
- Main UTI code + either Group C Structural abnormality/obstruction code or procedure within ±30 days of Main UTI code and/or Group A code within ±30 days of Main UTI code
  1. Consider as uUTI if positive urine culture with a UTI diagnosis code ±7 days of culture date, with the UTI diagnosis code being one of the following:
- Main UTI code + no Group C Structural abnormality/obstruction code or procedure within ±30 days of Main UTI code + no Group A code within ±30 days of Main UTI code

**Group A: cUTI codes**

| **ICD-10 CM** | **Code Description** |
| --- | --- |
| N11.0 | Nonobstructive reflux-associated chronic pyelonephritis |
| N11.1 | Chronic obstructive pyelonephritis |
| N13.6 | Pyonephrosis |
| N99.511 | Cystostomy infection |
| T83.510A | Infection and inflammatory reaction due to cystostomy catheter, initial encounter |
| T83.511A | Infection and inflammatory reaction due to indwelling urethral catheter, initial encounter |
| T83.512A | Infection and inflammatory reaction due to nephrostomy catheter, initial encounter |
| T83.518A | Infection and inflammatory reaction due to other urinary catheter, initial encounter |
| **ICD-9 CM** |  |
| 596.81 | Infection of cystostomy |
| 996.64 | Infection and inflammatory reaction due to indwelling urinary catheter |

Occurrence of these codes on their own is considered cUTI.

ICD-9-CM, International Classification of Diseases, Ninth Revision, Clinical Modification; ICD-10 CM: International Classification of Diseases, Tenth Revision, Clinical Modification

*Changes from the Carreno et al. 2019: added N11.1, N13.6, 596.81; moved from Group A to Group B: N10, N11.8, N12, N15.1, N15.9, N16, N28.84, N28.85, N28.86, 590.00, 590.01, 590.2, 590.3, 590.80, 590.9; moved from Group A to Group C: N35.111, N35.112, N35.113, N35.114, N35.116, N35.119, N35.12, 598.00, 598.01; moved from Group C to Group A: N99.511; removed 590.81

**Group B: Infection codes**

| **ICD-10 CM** | **Code Description** |
| --- | --- |
| N10 | Acute pyelonephritis |
| N11.8 | Other chronic tubulo-interstitial nephritis |
| N12 | Tubulo-interstitial nephritis, not specified as acute or chronic |
| N15.1 | Renal and perinephric abscess |
| N15.9 | Renal tubulo-interstitial disease, unspecified |
| N28.84 | Pyelitis cystica |
| N28.85 | Pyeloureteritis cystica |
| N28.86 | Ureteritis cystica |
| N30.00 | Acute cystitis without hematuria |
| N30.01 | Acute cystitis with hematuria |
| N30.10 | Interstitial cystitis (chronic) without hematuria |
| N30.11 | Interstitial cystitis (chronic) with hematuria |
| N30.20 | Other chronic cystitis without hematuria |
| N30.21 | Other chronic cystitis with hematuria |
| N30.30 | Trigonitis without hematuria |
| N30.31 | Trigonitis with hematuria |
| N30.80 | Other cystitis without hematuria |
| N30.81 | Other cystitis with hematuria |
| N30.90 | Cystitis, unspecified without hematuria |
| N30.91 | Cystitis, unspecified with hematuria |
| N34.0 | Urethral abscess |
| N34.1 | Urethritis, unspecified |
| N34.2 | Other urethritis |
| N39.0 | Urinary tract infection, site not specified |
| **ICD-9 CM** |  |
| 590.00 | Chronic pyelonephritis without lesion of renal medullary necrosis |
| 590.01 | Chronic pyelonephritis with lesion of renal medullary necrosis |
| 590.10 | Acute pyelonephritis with lesion of renal medullary necrosis |
| 590.11 | Acute pyelonephritis with lesion of renal medullary necrosis |
| 590.2 | Renal and perinephric abscess |
| 590.3 | Pyeloureteritis cystica |
| 590.80 | Other pyelonephritis or pyonephrosis, not specified as acute or chronic |
| 590.9 | Infection of kidney, unspecified |
| 595.0 | Acute cystitis |
| 595.1 | Chronic interstitial cystitis |
| 595.2 | Other chronic cystitis |
| 595.3 | Trigonitis |
| 595.4 | Cystitis in diseases classified elsewhere |
| 595.81 | Cystitis cystica |
| 595.89 | Other specified types of cystitis |
| 595.9 | Cystitis, unspecified |
| 597.0 | Urethral abscess |
| 597.80 | Urethritis, unspecified |
| 597.89 | Other urethritis |
| 599.0 | Urinary tract infection, site not specified |

Occurrence of these codes plus a Group C code within ≤30 days is considered cUTI. Shaded rows are also the Main UTI codes.

ICD-9-CM, International Classification of Diseases, Ninth Revision, Clinical Modification; ICD-10 CM: International Classification of Diseases, Tenth Revision, Clinical Modification

*Change from the Carreno et al. 2019: moved from Group A to Group B: N10, N11.8, N12, N15.1, N15.9, N16 (removed), N28.84, N28.85, N28.86, 590.00, 590.01, 590.2, 590.3, 590.80, 590.9; moved from Group B to Group C: N13.9, N35.014, N35.028, N35.811, N35.812, N35.813, N35.814, N35.816, N35.819, N35.82, N35.91, N35.92, N35.911, N35.912, N35.913, N35.914, N35.916, N35.919, N36.0, N36.1, N36.2, N36.5, N99.110, 598.00, 598.01, 598.1, 598.2, 598.8, 598.9, 599.1, 599.2, 599.3, 599.4, 599.5, 599.60, 599.69; removed N30.40, N30.41, N34.3, N36.8, N37, 595.82, 597.81

**Group C: Structural abnormality/obstruction codes**

| **ICD-10 CM** | **Code Description** |
| --- | --- |
| N13.0 | Hydronephrosis with ureteropelvic junction obstruction |
| N13.1 | Hydronephrosis with ureteral stricture, NEC |
| N13.2 | Hydronephrosis with renal and ureteral calculous obstruction |
| N13.30 | Unspecified hydronephrosis |
| N13.39 | Other hydronephrosis |
| N13.4 | Hydroureter |
| N13.5 | Crossing vessel and stricture of ureter without hydronephrosis |
| N13.70 | Vesicoureteral reflux, unspecified |
| N13.71 | Vesicoureteral reflux without reflux nephropathy |
| N13.721 | Vesicoureteral reflux with reflux nephropathy without hydroureter, unilateral |
| N13.722 | Vesicoureteral reflux with reflux nephropathy without hydroureter, bilateral |
| N13.729 | Vesicoureteral reflux with reflux nephropathy without hydroureter, unspecified |
| N13.731 | Vesicoureteral reflux with reflux nephropathy with hydroureter, unilateral |
| N13.732 | Vesicoureteral reflux with reflux nephropathy with hydroureter, bilateral |
| N13.739 | Vesicoureteral reflux with reflux nephropathy with hydroureter, unspecified |
| N13.8 | Other obstructive and reflux uropathy |
| N13.9 | Obstructive and reflux uropathy, unspecified |
| N20.0 | Calculus of the kidney |
| N20.1 | Calculus of ureter |
| N20.2 | Calculus of kidney with calculus of ureter |
| N20.9 | Urinary calculus, unspecified |
| N28.82 | Megaloureter |
| N28.89 | Other specified disorders of kidney and ureter |
| N31.2 | Flaccid neuropathic bladder, NEC |
| N31.9 | Neuromuscular dysfunction of bladder, unspecified |
| N32.0 | Bladder neck obstruction |
| N32.89 | Other specified disorders of bladder |
| N32.9 | Unspecified disorder of bladder |
| N35.010 | Post-traumatic urethral stricture, male, meatal |
| N35.011 | Post-traumatic bulbous urethral stricture, male |
| N35.012 | Post-traumatic membranous urethral stricture, male |
| N35.013 | Post-traumatic anterior urethral stricture, male |
| N35.014 | Post-traumatic urethral stricture, male, unspecified |
| N35.016 | Post-traumatic urethral stricture, male, overlapping sites |
| N35.021 | Urethral stricture due to childbirth, female |
| N35.028 | Other post-traumatic urethral stricture, female |
| N35.111 | Postinfective urethral stricture, NEC, male, meatal |
| N35.112 | Postinfective bulbous urethral stricture, NEC, male |
| N35.113 | Postinfective membranous urethral stricture, NEC, male |
| N35.114 | Postinfective anterior urethral stricture, NEC, male |
| N35.116 | Postinfective urethral stricture, NEC, male, overlapping sites |
| N35.119 | Postinfective urethral stricture, NEC, male, unspecified |
| N35.12 | Postinfective urethral stricture, NEC, female |
| N35.811 | Other urethral stricture, male, meatal |
| N35.812 | Other urethral bulbous stricture, male |
| N35.813 | Other membranous urethral stricture, male |
| N35.814 | Other anterior urethral stricture, male |
| N35.816 | Other urethral stricture, male, overlapping sites |
| N35.819 | Other urethral stricture, male, unspecified site |
| N35.82 | Other urethral stricture, female |
| N35.911 | Unspecified urethral stricture, male, meatal |
| N35.912 | Unspecified bulbous urethral stricture, male |
| N35.913 | Unspecified membranous urethral stricture, male |
| N35.914 | Unspecified anterior urethral stricture, male |
| N35.916 | Unspecified urethral stricture, male, overlapping sites |
| N35.919 | Unspecified urethral stricture, male, unspecified site |
| N35.92 | Unspecified urethral stricture, female |
| N36.0 | Urethral fistula |
| N36.1 | Urethral diverticulum |
| N36.2 | Urethral caruncle |
| N36.44 | Muscular disorders of urethra |
| N36.5 | Urethral false passage |
| N36.8 | Other specified disorders of urethra |
| N40.0 | Benign prostatic hyperplasia without lower urinary tract symptoms |
| N40.1 | Benign prostatic hyperplasia with lower urinary tract symptoms |
| N40.2 | Nodular prostate without lower urinary tract symptoms |
| N40.3 | Nodular prostate with lower urinary tract symptoms |
| N42.83 | Cyst of prostate |
| N99.110 | Postprocedural urethral stricture, male, meatal |
| N99.510 | Cystostomy hemorrhage |
| N99.512 | Cystostomy malfunction |
| N99.518 | Other complication of cystostomy |
| Q62.10 | Congenital occlusion of ureter, unspecified |
| Q62.11 | Congenital occlusion of ureteropelvic junction |
| Q62.12 | Congenital occlusion of ureterovesical orifice |
| Q62.31 | Congenital ureterocele, orthotopic |
| Q62.39 | Other obstructive defects of renal pelvis and ureter |
| R33.8 | Other retention of urine |
| R33.9 | Retention of urine, unspecified |
| R39.14 | Feeling of incomplete bladder emptying |
| Z43.6 | Encounter for attention to other artificial openings of urinary tract |
| Z46.6 | Encounter for fitting and adjustment of urinary device |
| Z96.0 | Presence of urogenital implants |
| **ICD-9-CM** |  |
| 591 | Hydronephrosis |
| 592.0 | Calculus of kidney |
| 592.1 | Calculus of ureter |
| 592.9 | Urinary calculus, unspecified |
| 593.3 | Stricture or kinking of ureter |
| 593.4 | Other ureteric obstruction |
| 593.5 | Hydroureter |
| 593.70 | Vesicoureteral reflux unspecified or without reflux nephropathy |
| 593.71 | Vesicoureteral reflux with reflux nephropathy, unilateral |
| 593.72 | Vesicoureteral reflux with reflux nephropathy, bilateral |
| 593.73 | Other vesicoureteral reflux with reflux nephropathy NOS |
| 593.82 | Ureteral fistula |
| 593.89 | Other specified disorders of kidney and ureter |
| 596.0 | Bladder neck obstruction |
| 596.4 | Atony of bladder |
| 596.53 | Paralysis of bladder |
| 596.54 | Neurogenic bladder |
| 596.55 | Detrusor sphincter dyssynergia |
| 596.82 | Mechanical complication of cystostomy |
| 596.83 | Other complication of cystostomy |
| 598.00 | Urethral stricture due to unspecified infection |
| 598.01 | Urethral stricture due to infective diseases classified elsewhere |
| 598.1 | Traumatic urethral stricture |
| 598.2 | Postoperative urethral stricture |
| 598.8 | Other specified causes of urethral stricture |
| 598.9 | Urethral stricture, unspecified |
| 599.1 | Urethral fistula |
| 599.2 | Urethral diverticulum |
| 599.3 | Urethral caruncle |
| 599.4 | Urethral false passage |
| 599.5 | Prolapse urethral mucosa |
| 599.60 | Urinary obstruction, unspecified |
| 599.69 | Urinary obstruction, not elsewhere classified |
| 600.00 | Hypertrophy (benign) of prostate without urinary obstruction and other lower urinary tract symptoms |
| 600.01 | Hypertrophy (benign) of prostate with urinary obstruction and other lower urinary tract symptoms |
| 600.10 | Nodular prostate without urinary obstruction |
| 600.11 | Nodular prostate with urinary obstruction |
| 600.20 | Benign localized hyperplasia of prostate without urinary obstruction and other lower urinary tract symptoms |
| 600.21 | Benign localized hyperplasia of prostate with urinary obstruction and other lower urinary tract symptoms |
| 600.3 | Cyst of prostate |
| 600.90 | Hyperplasia of prostate, unspecified, without urinary obstruction and other lower urinary symptoms |
| 600.91 | Hyperplasia of prostate, unspecified, with urinary obstruction and other lower urinary symptoms |
| 753.20 | Unspecified obstructive defect of renal pelvis and ureter |
| 753.21 | Congenital obstruction of ureteropelvic junction |
| 753.22 | Congenital obstruction of ureterovesical junction |
| 753.23 | Congenital ureterocele |
| 753.29 | Other obstructive defects of renal pelvis and ureter |
| 788.20 | Retention of urine, unspecified |
| 788.21 | Incomplete bladder emptying |
| 788.29 | Other specified retention of urine |
| 996.76 | Other complications due to genitourinary device, implant and graft |
| V53.6 | Fitting and adjustment of urinary devices |
| V55.6 | Attention to other artificial opening of urinary tract |

Occurrence of these codes plus a Group B code within ≤30 days is considered cUTI.

ICD-10 CM: International Classification of Diseases, Tenth Revision, Clinical Modification; NEC: not elsewhere classified; not otherwise specified

*Change from Carreno et al. 2019: added N13.0, N13.1, N13.2, N13.4, N13.5, N13.70, N13.71, N13.721, N13.722, N13.729, N13.731, N13.732, N13.739, N13.8, N28.82, N28.89, N35.010, N35.011, N35.012, N35.013, N35.016, N35.021, N36.8, Z96.0, 593.3, 593.4, 593.5, 593.71, 593.72, 593.73, 593.74, 593.82, 593.89, 596.82, 596.83, 996.76; moved from Group A to Group C: N35.111, N35.112, N35.113, N35.114, N35.116, N35.119, N35.12, 598.00, 598.01; moved from Group B to Group C: N13.9, N35.014, N35.028, N35.811, N35.812, N35.813, N35.814, N35.816, N35.819, N35.82, N35.91, N35.92, N35.911, N35.912, N35.913, N35.914, N35.916, N35.919, N36.0, N36.1, N36.2, N36.5, N99.110, 598.00, 598.01, 598.1, 598.2, 598.8, 598.9, 599.1, 599.2, 599.3, 599.4, 599.5, 599.60, 599.69; moved from Group C to Group A: N99.511

**Group C: procedures**

| **Code** | **Code Description** |
| --- | --- |
| **CPT-4** |  |
| 51702 | Insertion of temporary indwelling bladder catheter, simple (e.g., Foley) |
| 51703 | Insertion of temporary indwelling bladder catheter, complicated (e.g., altered anatomy, fractured catheter/balloon) |
| **ICD-10, PCS** |  |
| 0T2BX0Z | Change drainage device in bladder, external approach |
| 3C1ZX8Z | Irrigation of indwelling device using irrigating substance, external approach |
| **ICD-9, PCS** |  |
| 57.94 | Insertion of indwelling urinary catheter |
| 59.93 | Replacement of ureterostomy tube |
| 59.94 | Replacement of cystostomy tube |
| 57.95 | Replacement of indwelling urinary catheter |
| 96.45 | Irrigation of nephrostomy and pyelostomy |
| 96.46 | Irrigation of ureterostomy and ureteral catheter |
| 96.47 | Irrigation of cystostomy |
| 96.48 | Irrigation of indwelling urinary catheter |

Occurrence of these codes plus a Group B code within ≤30 days is considered cUTI.

CPT-4: Current Procedural Terminology, 4^th^ edition; ICD-9, PCS: International Classification of Diseases, Ninth Revision, Procedure Coding System;

ICD-10, PCS: International Classification of Diseases, Tenth Revision, Procedure Coding System

*Change from Carreno et al. 2019: removed 0T9B70Z, 0T9B80Z; added 59.93, 59.94, 96.45, 96.46, 96.47

| **STable 1. Baseline characteristics of individuals in the UTI cohort, with and without rUTI, Kaiser Permanente Southern California, 2016-2021.** | | | | | | | |
| --- | --- | --- | --- | --- | --- | --- | --- |
|  | **rUTI** | | **No rUTI** | | **Total** | |  |
|  | **N=62,277** | | **N=379,574** | | **N=441,851** | |  |
|  | **n** | **column %** | **n** | **column %** | **n** | **column %** | **p-value** |
| **Age group, years^1^** |  |  |  |  |  |  | <0.001 |
| 18-27 | 11,702 | 18.8 | 68,213 | 18.0 | 79,915 | 18.1 | 18-27 |
| 28-37 | 10,295 | 16.5 | 67,921 | 17.9 | 78,216 | 17.7 | 28-37 |
| 38-47 | 8,844 | 14.2 | 62,403 | 16.4 | 71,247 | 16.1 | 38-47 |
| 48-57 | 8,835 | 14.2 | 62,502 | 16.5 | 71,337 | 16.1 | 48-57 |
| 58-67 | 8,912 | 14.3 | 55,467 | 14.6 | 64,379 | 14.6 | 58-67 |
| 68-77 | 7,499 | 12.0 | 38,661 | 10.2 | 46,160 | 10.4 | 68-77 |
| ≥78 | 6,190 | 9.9 | 24,407 | 6.4 | 30,597 | 6.9 | ≥78 |
| **Age, years, median [IQR]** | 48.0  (31.0-66.0) |  | 46.0  (31.0-61.0) |  | 46.0  (31.0-62.0) |  |  |
| **Sex** |  |  |  |  |  |  | <0.001 |
| Female | 59,459 | 95.5 | 348,020 | 91.7 | 407,479 | 92.2 |  |
| Male | 2,816 | 4.5 | 31,547 | 8.3 | 34,363 | 7.8 |  |
| Other | 2 | 0.0 | 7 | 0.0 | 9 | 0.0 |  |
| **Race/ethnicity** |  |  |  |  |  |  | <0.001 |
| Asian/ Pacific Islander | 5,204 | 8.4 | 36,264 | 9.6 | 41,468 | 9.4 |  |
| Black | 4,715 | 7.6 | 32,778 | 8.6 | 37,493 | 8.5 |  |
| Hispanic | 27,227 | 43.7 | 171,879 | 45.3 | 199,106 | 45.1 |  |
| White | 23,317 | 37.4 | 126,838 | 33.4 | 150,155 | 34.0 |  |
| Other/ Unknown | 1,814 | 2.9 | 11,815 | 3.1 | 13,629 | 3.1 |  |
| **Body mass index^1^** |  |  |  |  |  |  | <0.001 |
| <18.5 | 1,173 | 1.9 | 5,962 | 1.6 | 7,135 | 1.6 |  |
| 18.5-24.9 | 18,890 | 30.3 | 102,528 | 27.0 | 121,418 | 27.5 |  |
| 25.0-29.9 | 17,128 | 27.5 | 103,635 | 27.3 | 120,763 | 27.3 |  |
| 30.0-39.9 | 16,280 | 26.1 | 101,062 | 26.6 | 117,342 | 26.6 |  |
| ≥40.0 | 4,023 | 6.5 | 25,221 | 6.6 | 29,244 | 6.6 |  |
| Unknown | 4,783 | 7.7 | 41,166 | 10.9 | 45,949 | 10.4 |  |
| **Smoking status^2^** |  |  |  |  |  |  | <0.001 |
| Never | 47,448 | 76.2 | 291,674 | 76.8 | 339,122 | 76.8 |  |
| Ever | 135,84 | 21.8 | 78,937 | 20.8 | 92,521 | 20.9 |  |
| Unknown | 1,245 | 2.0 | 8,963 | 2.4 | 10,208 | 2.3 |  |
| **Neighborhood-level income** |  |  |  |  |  |  | <0.001 |
| <$40,000 | 2,443 | 3.9 | 15,084 | 4.0 | 17,527 | 4.0 |  |
| $40,000 - <$60,000 | 11,211 | 18.0 | 71,463 | 18.8 | 82,674 | 18.7 |  |
| $60,000 - <$85,000 | 18,931 | 30.4 | 116,906 | 30.8 | 135,837 | 30.7 |  |
| ≥$85,000 | 29,337 | 47.1 | 174,260 | 45.9 | 203,597 | 46.1 |  |
| Unknown | 355 | 0.6 | 1,861 | 0.50 | 2,216 | 0.5 |  |
| **Medicaid** | 6,853 | 11.0 | 36,104 | 9.5 | 42,957 | 9.7 | <0.001 |
| **Charlson Comorbidity Index score^2^** |  |  |  |  |  |  | <0.001 |
| 0 | 36,990 | 59.4 | 249,768 | 65.8 | 286,758 | 64.9 |  |
| 1-2 | 19,595 | 31.5 | 108,076 | 28.5 | 127,671 | 28.9 |  |
| ≥3 | 5,692 | 9.1 | 21,730 | 5.7 | 27,422 | 6.2 |  |
| **Diabetes^2^** | 10,372 | 16.7 | 52,633 | 13.9 | 63,005 | 14.3 | <0.001 |
| **Renal disease^2^** | 5,312 | 8.5 | 22,908 | 6.0 | 28,220 | 6.4 | <0.001 |
| **Dementia^2^** | 1,551 | 2.5 | 5,239 | 1.4 | 6,790 | 1.5 | <0.001 |
| **Immunocompromised^1^** | 1,902 | 3.1 | 7,959 | 2.1 | 9,861 | 2.2 | <0.001 |
| **Pregnancy^2^** | 885 | 1.4 | 6,904 | 1.8 | 7,789 | 1.8 | <0.001 |
| **Number of outpatient visits^2^** |  |  |  |  |  |  | <0.001 |
| 0 | 2,011 | 3.2 | 21,013 | 5.5 | 23,024 | 5.2 |  |
| 1-4 | 15,660 | 25.2 | 120,549 | 31.8 | 136,209 | 30.8 |  |
| 5-8 | 14,574 | 23.4 | 90,294 | 23.8 | 104,868 | 23.7 |  |
| 9-15 | 14,730 | 23.7 | 78,875 | 20.8 | 93,605 | 21.2 |  |
| $\geq$16 | 15,302 | 24.6 | 68,843 | 18.1 | 84,145 | 19.0 |  |
| **Number of emergency department visits^2^** |  |  |  |  |  |  | <0.001 |
| 0 | 47,354 | 76.0 | 304,970 | 80.4 | 352,324 | 79.7 |  |
| 1 | 9,838 | 15.8 | 52,503 | 13.8 | 62,341 | 14.1 |  |
| ≥2 | 5,085 | 8.2 | 22,101 | 5.8 | 27,186 | 6.2 |  |
| **Number of inpatient visits^1^** |  |  |  |  |  |  | <0.001 |
| 0 | 56,420 | 90.6 | 350,518 | 92.4 | 406,938 | 92.1 |  |
| 1 | 4,448 | 7.1 | 23,474 | 6.2 | 27,922 | 6.3 |  |
| ≥2 | 1,409 | 2.3 | 5,582 | 1.5 | 6,991 | 1.6 |  |
| **Number of antibiotic prescriptions^2^** |  |  |  |  |  |  | <0.001 |
| 0 | 36,383 | 58.4 | 254,034 | 67.0 | 290,417 | 65.7 |  |
| 1-2 | 19,428 | 31.2 | 101,661 | 26.8 | 121,089 | 27.4 |  |
| ≥3 | 6,466 | 10.4 | 23,879 | 6.3 | 30,345 | 6.9 |  |
| **Antibiotic prescriptions class^2,3^** |  |  |  |  |  |  |  |
| Aminoglycoside | 358 | 0.6 | 1,866 | 0.5 | 2,224 | 0.5 | 0.007 |
| Carbapenem | 64 | 0.1 | 178 | 0.1 | 242 | 0.1 | 0.001 |
| Cephalosporin | 13,327 | 21.4 | 59,600 | 15.7 | 72,927 | 16.5 | <0.001 |
| Fluoroquinolone | 4,782 | 7.7 | 17,211 | 4.5 | 21,993 | 5.0 | <0.001 |
| Fosfomycin | 18 | 0.0 | 34 | 0.0 | 52 | 0.0 | <0.001 |
| Nitrofurantoin | 2,485 | 4.0 | 6,510 | 1.7 | 8,995 | 2.0 | <0.001 |
| Penicillin | 11,509 | 18.5 | 61,391 | 16.2 | 72,900 | 16.5 | <0.001 |
| Trimethoprim-sulfamethoxazole | 3,103 | 5 | 12,807 | 3.4 | 15,910 | 3.6 | <0.001 |
| Others | 777 | 1.3 | 3,137 | 0.8 | 3,914 | 0.90 | <0.001 |
| **Index uUTI type** |  |  |  |  |  |  | <0.001 |
| Pyelonephritis | 511 | 0.8 | 3,493 | 0.9 | 4,004 | 0.9 |  |
| Cystitis | 55,798 | 89.6 | 343,205 | 90.4 | 399,003 | 90.3 |  |
| Others | 5,968 | 9.6 | 32,876 | 8.7 | 38,844 | 8.8 |  |
| **Year of index uUTI** |  |  |  |  |  |  | <0.001 |
| 2016 | 13,668 | 22.0 | 81,021 | 21.4 | 94,689 | 21.4 |  |
| 2017 | 12,904 | 20.7 | 79,712 | 21.0 | 92,616 | 21.0 |  |
| 2018 | 12,935 | 20.8 | 77,915 | 20.5 | 90,850 | 20.6 |  |
| 2019 | 11,650 | 18.7 | 73,955 | 19.5 | 85,605 | 19.4 |  |
| 2020 | 11,120 | 17.9 | 66,971 | 17.6 | 78,091 | 17.7 |  |
| **Urine culture at index uUTI** |  |  |  |  |  |  | <0.001 |
| No culture | 32,127 | 51.6 | 195,310 | 51.5 | 227,437 | 51.5 |  |
| Negative urine culture | 6,407 | 10.3 | 58,135 | 15.3 | 64,542 | 14.6 |  |
| Positive urine culture | 23,743 | 38.1 | 126,129 | 33.2 | 149,872 | 33.9 |  |
|  |  |  |  |  |  |  |  |
| **Number with positive urine culture^4^** | **23,743** |  | **126,129** |  | **149,872** |  |  |
| ***E. coli* isolated at index uUTI, among those with positive cultures^5^** | 18,764 | 79.0 | 101,579 | 80.5 | 120,343 | 80.3 | <0.001 |
| ***Klebsiella spp* isolated at index uUTI, among those with positive cultures^5^** | 1,998 | 8.4 | 8,374 | 6.6 | 10,372 | 6.9 | <0.001 |
| **Non-susceptibility to antibiotic classes among those with positive cultures^6^** |  |  |  |  |  |  | <0.001 |
| Susceptible | 9,618 | 40.5 | 54,447 | 43.2 | 64,065 | 42.8 |  |
| Non-susceptible to 1-2 antibiotic classes | 10,630 | 44.8 | 55,704 | 44.2 | 66,334 | 44.3 |  |
| Non-susceptible to ≥3 antibiotic classes | 3,406 | 14.4 | 15,189 | 12.0 | 18,595 | 12.4 |  |
| Unknown | 89 | 0.4 | 789 | 0.6 | 878 | 0.6 |  |
| Abbreviations: rUTI, recurrent urinary tract infection; uUTI, uncomplicated urinary tract infection; IQR, interquartile range | | | | | | | |
| ^1^At index date (date of the earliest record for the index uUTI) | | | | | | | |
| ^2^In the year before index date | | | | | | | |
| ^3^Not mutually exclusive | | | | | | | |
| ^4^Excludes cultures with unknown susceptibility | | | | | | | |
| ^5^Other organisms isolated included *Proteus mirabilis* (4.29%), *Citrobacter spp* (1.59%), *Enterobacter spp* (1.51%), *Enterococcus spp* (0.92%), *Pseudomonas aeruginosa* (0.46%), Coagulase-negative *Staphylococcus spp* (0.32%). More than one pathogen was isolated from 1.83% of positive cultures. | | | | | | | |
| ^6^Antibiotic classes were aminoglycosides, carbapenems, cephalosporins, fluroquinolones, fosfomycin, nitrofurantoin, penicillin, trimethoprim-sulfamethoxazole, others (aztreonam, colistin, daptomycin, linezolid, tigecycline, vancomycin) | | | | | | | |

| **STable 2.** **Factors associated with rUTI among women with cystitis as index uUTI, by race/ethnicity** | | | | | |
| --- | --- | --- | --- | --- | --- |
|  | **White** | **Black** | **Hispanic** | **Asian/ Pacific Islander** | **Other/ Unknown** |
|  | **Adjusted RR (95% CI)^1^** | **Adjusted RR (95% CI)^1^** | **Adjusted RR (95% CI)^1^** | **Adjusted RR (95% CI)^1^** | **Adjusted RR (95% CI)^1^** |
| **Age group (years)^2^** |  |  |  |  |  |
| 18-27 | 1.00 | 1.00 | 1.00 | 1.00 | 1.00 |
| 28-37 | 0.88 (0.84 - 0.93) | 0.91 (0.83 - 1.00) | 0.94 (0.91 - 0.98) | 0.88 (0.81 - 0.96) | 0.78 (0.69 - 0.88) |
| 38-47 | 0.88 (0.84 - 0.93) | 0.88 (0.80 - 0.97) | 0.87 (0.83 - 0.90) | 0.77 (0.70 - 0.84) | 0.79 (0.69 - 0.91) |
| 48-57 | 0.84 (0.80 - 0.89) | 0.77 (0.69 - 0.86) | 0.84 (0.81 - 0.88) | 0.76 (0.69 - 0.84) | 0.74 (0.64 - 0.87) |
| 58-67 | 0.91 (0.86 - 0.95) | 0.71 (0.63 - 0.80) | 0.91 (0.87 - 0.95) | 0.73 (0.66 - 0.81) | 0.83 (0.70 - 0.98) |
| 68-77 | 0.98 (0.93 - 1.03) | 0.88 (0.77 - 1.00) | 0.98 (0.93 - 1.03) | 0.73 (0.65 - 0.83) | 0.75 (0.59 - 0.96) |
| ≥78 | 1.11 (1.05 - 1.17) | 0.88 (0.76 - 1.01) | 1.12 (1.04 - 1.19) | 0.89 (0.77 - 1.03) | 1.20 (0.89 - 1.63) |
| **Body mass index^3^** |  |  |  |  |  |
| <18.5 | 1.00 | 1.00 | 1.00 | 1.00 | 1.00 |
| 18.5-24.9 | 1.00 (0.92 - 1.09) | 1.19 (0.95 - 1.51) | 0.98 (0.88 - 1.09) | 1.09 (0.94 - 1.27) | 0.98 (0.74 - 1.31) |
| 25.0-29.9 | 0.94 (0.87 - 1.03) | 1.08 (0.85 - 1.36) | 0.90 (0.81 - 1.01) | 1.02 (0.87 - 1.19) | 0.91 (0.68 - 1.22) |
| 30.0-39.9 | 0.90 (0.82 - 0.98) | 1.05 (0.84 - 1.33) | 0.88 (0.79 - 0.98) | 0.98 (0.83 - 1.16) | 0.81 (0.60 - 1.09) |
| ≥40.0 | 0.90 (0.82 - 0.99) | 0.97 (0.76 - 1.24) | 0.82 (0.73 - 0.92) | 1.17 (0.90 - 1.51) | 0.87 (0.62 - 1.23) |
| Unknown | 1.02 (0.92 - 1.13) | 1.03 (0.79 - 1.35) | 0.90 (0.79 - 1.01) | 1.05 (0.87 - 1.26) | 0.88 (0.64 - 1.21) |
| **Neighborhood-level income** | | | | | |
| <$40,000 | 1.00 | 1.00 | 1.00 | 1.00 | 1.00 |
| $40,000 - <$60,000 | 0.96 (0.87 - 1.05) | 1.03 (0.92 - 1.16) | 0.99 (0.93 - 1.05) | 1.13 (0.90 - 1.41) | 0.77 (0.60 - 0.98) |
| $60,000 - <$85,000 | 1.01 (0.93 - 1.10) | 1.06 (0.94 - 1.18) | 1.01 (0.96 - 1.07) | 1.06 (0.85 - 1.31) | 0.80 (0.64 - 1.01) |
| ≥$85,000 | 1.01 (0.92 - 1.09) | 1.04 (0.93 - 1.17) | 1.05 (1.00 - 1.12) | 1.10 (0.89 - 1.36) | 0.78 (0.63 - 0.97) |
| Unknown | 1.06 (0.92 - 1.23) | 1.58 (1.15 - 2.18) | 1.09 (0.86 - 1.39) | 0.83 (0.47 - 1.48) | 0.42 (0.18 - 1.01) |
| **Medicaid** | 1.08 (1.02 - 1.14) | 1.15 (1.07 - 1.25) | 1.06 (1.02 - 1.09) | 1.05 (0.95 - 1.17) | 0.83 (0.67 - 1.04) |
| **Charlson Comorbidity Index score^3^** |  |  |  |  |  |
| 0 | 1.00 | 1.00 | 1.00 | 1.00 | 1.00 |
| 1-2 | 1.03 (1.00 - 1.07) | 1.02 (0.94 - 1.10) | 1.02 (0.99 - 1.06) | 1.00 (0.92 - 1.08) | 1.03 (0.90 - 1.18) |
| ≥3 | 1.13 (1.07 - 1.20) | 1.09 (0.94 - 1.26) | 1.14 (1.06 - 1.22) | 0.97 (0.83 - 1.15) | 1.26 (0.88 - 1.80) |
| **Comorbidities** |  |  |  |  |  |
| Diabetes^3^ | 1.08 (1.03 - 1.13) | 1.04 (0.95 - 1.15) | 1.07 (1.03 - 1.12) | 1.17 (1.06 - 1.29) | 1.05 (0.85 - 1.29) |
| Dementia^3^ | 1.19 (1.11 - 1.28) | 1.33 (1.12 - 1.58) | 1.17 (1.05 - 1.30) | 1.27 (1.00 - 1.62) | 1.00 (0.59 - 1.71) |
| Immunocompromised^2^ | 1.11 (1.04 - 1.19) | 1.18 (1.02 - 1.37) | 1.11 (1.03 - 1.20) | 1.17 (0.97 - 1.40) | 1.19 (0.83 - 1.71) |
| **Pregnancy^3^** | 0.63 (0.52 - 0.75) | 0.72 (0.56 - 0.93) | 0.67 (0.60 - 0.74) | 0.43 (0.30 - 0.62) | 0.88 (0.48 - 1.60) |
| **Oral contraceptives^3^** | 1.11 (1.06 - 1.17) | 1.06 (0.95 - 1.19) | 1.12 (1.08 - 1.17) | 1.16 (1.06 - 1.27) | 1.16 (1.02 - 1.32) |
| **Number of outpatient visits^3^** |  |  |  |  |  |
| 0 | 1.00 | 1.00 | 1.00 | 1.00 | 1.00 |
| 1-4 | 1.33 (1.21 - 1.46) | 1.24 (0.98 - 1.57) | 1.17 (1.07 - 1.27) | 1.16 (0.98 - 1.37) | 1.32 (1.03 - 1.71) |
| 5-8 | 1.48 (1.34 - 1.64) | 1.46 (1.14 - 1.86) | 1.37 (1.25 - 1.50) | 1.38 (1.15 - 1.66) | 1.49 (1.13 - 1.96) |
| 9-15 | 1.58 (1.42 - 1.75) | 1.59 (1.24 - 2.03) | 1.51 (1.38 - 1.66) | 1.39 (1.15 - 1.68) | 1.57 (1.18 - 2.08) |
| ≥16 | 1.70 (1.54 - 1.89) | 1.63 (1.27 - 2.09) | 1.63 (1.48 - 1.80) | 1.51 (1.24 - 1.83) | 1.75 (1.30 - 2.35) |
| **Number of emergency department visits^3^** |  |  |  |  |  |
| 0 | 1.00 | 1.00 | 1.00 | 1.00 | 1.00 |
| 1 | 1.01 (0.98 - 1.05) | 1.02 (0.94 - 1.10) | 1.02 (0.98 - 1.05) | 1.12 (1.03 - 1.21) | 0.94 (0.81 - 1.09) |
| ≥2 | 1.00 (0.95 - 1.06) | 1.14 (1.04 - 1.25) | 1.08 (1.03 - 1.13) | 1.18 (1.04 - 1.34) | 1.09 (0.86 - 1.39) |
| **Number of inpatient visits^3^** | | | | | |
| 0 | 1.00 | 1.00 | 1.00 | 1.00 | 1.00 |
| 1 | 0.89 (0.84 - 0.93) | 0.95 (0.86 - 1.06) | 0.79 (0.75 - 0.83) | 0.76 (0.66 - 0.87) | 0.78 (0.60 - 1.02) |
| ≥2 | 0.93 (0.85 - 1.01) | 0.88 (0.73 - 1.05) | 0.86 (0.78 - 0.95) | 0.90 (0.71 - 1.16) | 0.67 (0.37 - 1.21) |
| **Number of antibiotic prescriptions^3^** | | | | | |
| 0 | 1.00 | 1.00 | 1.00 | 1.00 | 1.00 |
| 1-2 | 1.06 (1.02 - 1.10) | 1.10 (1.02 - 1.20) | 1.09 (1.05 - 1.12) | 1.05 (0.97 - 1.14) | 1.04 (0.91 - 1.19) |
| ≥3 | 1.14 (1.07 - 1.21) | 1.32 (1.16 - 1.51) | 1.22 (1.16 - 1.30) | 1.19 (1.03 - 1.38) | 1.12 (0.87 - 1.44) |
| **Antibiotic class^3,4^** |  |  |  |  |  |
| Cephalosporin | 1.11 (1.07 - 1.16) | 0.99 (0.90 - 1.09) | 1.08 (1.04 - 1.12) | 1.11 (1.00 - 1.22) | 1.34 (1.14 - 1.56) |
| Fluoroquinolone | 1.27 (1.21 - 1.34) | 1.40 (1.24 - 1.57) | 1.29 (1.23 - 1.35) | 1.38 (1.23 - 1.55) | 1.38 (1.14 - 1.68) |
| Nitrofurantoin | 1.64 (1.54 - 1.75) | 1.54 (1.33 - 1.77) | 1.63 (1.54 - 1.72) | 1.82 (1.60 - 2.09) | 1.53 (1.27 - 1.84) |
| **Year of index uUTI** |  |  |  |  |  |
| 2016 | 1.00 | 1.00 | 1.00 | 1.00 | 1.00 |
| 2017 | 0.97 (0.93 - 1.01) | 0.95 (0.88 - 1.04) | 0.96 (0.92 - 0.99) | 1.04 (0.95 - 1.13) | 0.97 (0.82 - 1.15) |
| 2018 | 0.99 (0.96 - 1.03) | 0.94 (0.86 - 1.03) | 0.98 (0.95 - 1.02) | 1.10 (1.01 - 1.20) | 1.21 (1.04 - 1.41) |
| 2019 | 0.91 (0.88 - 0.95) | 0.91 (0.83 - 0.99) | 0.95 (0.92 - 0.99) | 1.12 (1.03 - 1.22) | 1.09 (0.93 - 1.27) |
| 2020 | 0.99 (0.95 - 1.03) | 0.95 (0.87 - 1.05) | 1.01 (0.97 - 1.05) | 1.15 (1.05 - 1.25) | 1.14 (0.98 - 1.33) |
| **Urine culture at index uUTI** | | | | | |
| No culture | 1.00 | 1.00 | 1.00 | 1.00 | 1.00 |
| Negative urine culture | 0.73 (0.70 - 0.76) | 0.78 (0.71 - 0.86) | 0.78 (0.75 - 0.81) | 0.75 (0.69 - 0.82) | 0.63 (0.54 - 0.75) |
| Positive urine culture | 1.12 (1.09 - 1.15) | 1.29 (1.21 - 1.37) | 1.16 (1.13 - 1.19) | 1.04 (0.98 - 1.11) | 1.03 (0.93 - 1.14) |
| Abbreviations: CI, confidence interval; RR, risk ratio; rUTI, recurrent urinary tract infection; uUTI, uncomplicated urinary tract infection | | | | | |
| ^1^Adjusted RR and 95% CIs were estimated from modified Poisson regression models with robust error variance, adjusted for all other variables in the table. | | | | | |
| ^2^At index date (date of earliest record for the index uUTI). | | | | | |
| ^3^In the year prior to index date. | | | | | |
| ^4^For each antibiotic class, the comparison group did not receive an antibiotic of that class in the prior year. | | | | | |

| **STable 3. Factors associated with rUTI among women with cystitis as index uUTI with positive urine culture, by race/ethnicity** | | | | | |
| --- | --- | --- | --- | --- | --- |
|  | **White** | **Black** | **Hispanic** | **Asian/ Pacific Islander** | **Other/ Unknown** |
|  | **Adjusted RR (95% CI)^1^** | **Adjusted RR (95% CI)^1^** | **Adjusted RR (95% CI)^1^** | **Adjusted RR (95% CI)^1^** | **Adjusted RR (95% CI)^2^** |
| **Age group (years)^3^** |  |  |  |  |  |
| 18-27 | 1.00 | 1.00 | 1.00 | 1.00 | 1.00 |
| 28-37 | 0.85 (0.77 - 0.94) | 0.82 (0.68 - 1.00) | 0.91 (0.85 - 0.98) | 0.74 (0.62 - 0.88) | 0.80 (0.61 - 1.04) |
| 38-47 | 0.81 (0.73 - 0.90) | 0.82 (0.67 - 1.00) | 0.85 (0.79 - 0.92) | 0.72 (0.60 - 0.86) | 0.79 (0.60 - 1.05) |
| 48-57 | 0.85 (0.77 - 0.94) | 0.72 (0.59 - 0.88) | 0.83 (0.77 - 0.90) | 0.70 (0.58 - 0.83) | 0.88 (0.67 - 1.17) |
| 58-67 | 0.93 (0.85 - 1.02) | 0.68 (0.55 - 0.85) | 0.93 (0.85 - 1.01) | 0.70 (0.58 - 0.84) | 0.95 (0.71 - 1.26) |
| 68-77 | 1.02 (0.93 - 1.12) | 0.88 (0.70 - 1.11) | 1.02 (0.93 - 1.11) | 0.71 (0.58 - 0.87) | 1.06 (0.76 - 1.49) |
| ≥78 | 1.16 (1.05 - 1.28) | 0.89 (0.69 - 1.14) | 1.19 (1.07 - 1.32) | 0.78 (0.62 - 0.99) | 1.52 (0.94 - 2.46) |
| **Body mass index^4^** |  |  |  |  |  |
| <18.5 | 1.00 | 1.00 | 1.00 | 1.00 |  |
| 18.5-24.9 | 1.00 (0.86 - 1.16) | 1.25 (0.81 - 1.93) | 1.03 (0.84 - 1.26) | 0.92 (0.73 - 1.16) |  |
| 25.0-29.9 | 0.97 (0.84 - 1.13) | 1.09 (0.70 - 1.69) | 0.97 (0.79 - 1.19) | 0.81 (0.63 - 1.03) |  |
| 30.0-39.9 | 0.96 (0.82 - 1.11) | 1.23 (0.80 - 1.90) | 0.99 (0.80 - 1.21) | 0.77 (0.59 - 1.01) |  |
| ≥40.0 | 0.99 (0.83 - 1.17) | 0.98 (0.61 - 1.55) | 1.00 (0.81 - 1.25) | 1.17 (0.75 - 1.82) |  |
| Unknown | 0.90 (0.75 - 1.09) | 1.38 (0.83 - 2.28) | 0.97 (0.77 - 1.22) | 0.79 (0.57 - 1.09) |  |
| **Neighborhood-level income** | | | | | |
| <$40,000 | 1.00 | 1.00 | 1.00 | 1.00 |  |
| $40,000 - <$60,000 | 1.08 (0.91 - 1.28) | 0.96 (0.77 - 1.18) | 1.00 (0.90 - 1.12) | 1.21 (0.81 - 1.82) |  |
| $60,000 - <$85,000 | 1.15 (0.97 - 1.35) | 0.98 (0.80 - 1.21) | 1.08 (0.97 - 1.21) | 1.10 (0.74 - 1.64) |  |
| ≥$85,000 | 1.14 (0.97 - 1.34) | 0.97 (0.79 - 1.20) | 1.14 (1.02 - 1.26) | 1.16 (0.79 - 1.71) |  |
| Unknown | 1.00 (0.76 - 1.33) | 1.33 (0.71 - 2.48) | 1.32 (0.90 - 1.93) | 1.05 (0.44 - 2.49) |  |
| **Medicaid** | 1.14 (1.03 - 1.26) | 1.19 (1.03 - 1.38) | 1.11 (1.05 - 1.18) | 1.24 (1.05 - 1.48) |  |
| **Charlson Comorbidity Index score^4^** |  |  |  |  |  |
| 0 | 1.00 |  |  |  |  |
| 1–2 | 1.08 (1.01 - 1.14) | 0.96 (0.82 - 1.11) | 1.04 (0.98 - 1.11) | 1.13 (0.98 - 1.30) |  |
| ≥3 | 1.18 (1.07 - 1.29) | 0.90 (0.69 - 1.17) | 1.12 (1.00 - 1.25) | 0.92 (0.70 - 1.21) |  |
| **Comorbidities** |  |  |  |  |  |
| Diabetes^4^ | 1.05 (0.98 - 1.12) | 1.11 (0.94 - 1.31) | 1.07 (1.00 - 1.14) | 1.19 (1.02 - 1.39) |  |
| Dementia^4^ | 1.16 (1.03 - 1.30) | 1.61 (1.21 - 2.14) | 1.10 (0.93 - 1.31) | 1.53 (1.08 - 2.18) |  |
| Immunocompromised^3^ | 1.14 (1.03 - 1.27) | 1.19 (0.91 - 1.54) | 1.12 (0.99 - 1.26) | 1.21 (0.90 - 1.62) |  |
| **Oral contraceptives^4^** | 1.20 (1.08 - 1.32) | 0.87 (0.68 - 1.11) | 1.10 (1.00 - 1.19) | 1.07 (0.88 - 1.29) | 1.28 (0.98 - 1.66) |
| **Number of outpatient visits^4^** |  |  |  |  |  |
| 0 | 1.00 | 1.00 | 1.00 | 1.00 |  |
| 1–4 | 1.32 (1.08 - 1.60) | 1.81 (1.14 - 2.86) | 1.11 (0.94 - 1.30) | 1.12 (0.81 - 1.55) |  |
| 5–8 | 1.44 (1.18 - 1.77) | 2.04 (1.27 - 3.28) | 1.29 (1.09 - 1.53) | 1.32 (0.94 - 1.86) |  |
| 9–15 | 1.56 (1.27 - 1.91) | 2.37 (1.47 - 3.82) | 1.44 (1.21 - 1.70) | 1.31 (0.92 - 1.86) |  |
| ≥16 | 1.73 (1.41 - 2.13) | 2.49 (1.53 - 4.04) | 1.60 (1.34 - 1.90) | 1.55 (1.08 - 2.22) |  |
| **Number of emergency department visits^4^** | | | |  |  |
| 0 | 1.00 | 1.00 | 1.00 | 1.00 |  |
| 1 | 0.97 (0.91 - 1.04) | 1.00 (0.86 - 1.16) | 1.04 (0.99 - 1.11) | 1.04 (0.90 - 1.20) |  |
| ≥2 | 1.04 (0.95 - 1.14) | 1.15 (0.97 - 1.36) | 1.12 (1.03 - 1.22) | 1.27 (1.03 - 1.57) |  |
| **Number of inpatient visits^4^** | | | |  |  |
| 0 | 1.00 | 1.00 | 1.00 | 1.00 |  |
| 1 | 0.92 (0.84 - 1.00) | 1.03 (0.84 - 1.25) | 0.78 (0.71 - 0.85) | 0.77 (0.61 - 0.97) |  |
| ≥2 | 0.97 (0.85 - 1.11) | 1.04 (0.77 - 1.40) | 0.90 (0.76 - 1.06) | 0.89 (0.59 - 1.34) |  |
| **Number of antibiotic prescriptions^4^** | | | | |  |
| 0 | 1.00 | 1.00 | 1.00 | 1.00 |  |
| 1–2 | 1.03 (0.96 - 1.09) | 1.11 (0.96 - 1.29) | 1.06 (1.00 - 1.13) | 1.03 (0.89 - 1.18) |  |
| ≥3 | 1.04 (0.94 - 1.16) | 1.33 (1.03 - 1.71) | 1.21 (1.09 - 1.34) | 1.12 (0.86 - 1.46) |  |
| **Antibiotic prescription class^4,5^** |  |  |  |  |  |
| Cephalosporin | 1.12 (1.04 - 1.20) | 1.03 (0.86 - 1.23) | 1.09 (1.02 - 1.17) | 1.17 (0.98 - 1.40) |  |
| Fluoroquinolone | 1.30 (1.19 - 1.41) | 1.42 (1.15 - 1.76) | 1.34 (1.24 - 1.46) | 1.37 (1.10 - 1.70) |  |
| Nitrofurantoin | 1.60 (1.41 - 1.81) | 1.41 (1.07 - 1.86) | 1.62 (1.45 - 1.80) | 1.73 (1.32 - 2.27) |  |
| **Year of index uUTI** |  |  |  |  |  |
| 2016 | 1.00 | 1.00 | 1.00 | 1.00 |  |
| 2017 | 0.94 (0.88 - 1.00) | 0.96 (0.82 - 1.12) | 0.97 (0.91 - 1.03) | 0.88 (0.76 - 1.01) |  |
| 2018 | 0.94 (0.88 - 1.00) | 1.00 (0.86 - 1.18) | 1.01 (0.95 - 1.08) | 0.96 (0.83 - 1.11) |  |
| 2019 | 0.85 (0.79 - 0.91) | 0.93 (0.78 - 1.09) | 0.91 (0.85 - 0.97) | 0.99 (0.86 - 1.14) |  |
| 2020 | 0.90 (0.83 - 0.97) | 0.98 (0.81 - 1.18) | 0.98 (0.91 - 1.06) | 1.03 (0.88 - 1.20) |  |
| **Urinary pathogens at index uUTI^6^** |  |  |  |  |  |
| ***E. coli*** |  |  |  |  |  |
| Not *E. coli* | 1.00 | 1.00 | 1.00 | 1.00 | 1.00 |
| *E. coli* isolated^7^ | 1.05 (0.98 - 1.13) | 1.02 (0.87 - 1.18) | 1.06 (0.99 - 1.14) | 0.99 (0.85 - 1.16) | 1.57 (1.09 - 2.25) |
| ***Klebsiella spp*** |  |  |  |  |  |
| Not *Klebsiella spp* | 1.00 | 1.00 | 1.00 | 1.00 | 1.00 |
| *Klebsiella spp* isolated^8^ | 1.14 (1.04 - 1.25) | 1.33 (1.08 - 1.63) | 1.24 (1.13 - 1.37) | 0.97 (0.76 - 1.24) | 1.60 (1.01 - 2.54) |
| **Non-susceptibility to antibiotics** |  |  |  |  |  |
| Susceptible | 1.00 | 1.00 | 1.00 | 1.00 | 1.00 |
| Non-susceptible to 1-2 antibiotic classes^9^ | 1.03 (0.98 - 1.08) | 1.04 (0.92 - 1.19) | 1.00 (0.95 - 1.05) | 1.02 (0.91 - 1.14) | 1.00 (0.82 - 1.21) |
| Non-susceptible to ≥3 antibiotic classes^9^ | 1.16 (1.08 - 1.24) | 1.32 (1.11 - 1.58) | 1.04 (0.98 - 1.11) | 1.15 (1.00 - 1.33) | 1.32 (1.02 - 1.72) |
| Abbreviations: CI, confidence interval; RR, risk ratio; rUTI, recurrent urinary tract infection; uUTI, uncomplicated urinary tract infection | | | | | |
| ^1^Adjusted RR and 95% confidence intervals (CIs) were estimated from modified robust Poisson regression models, adjusted for all other variables listed in table. | | | | | |
| ^2^Model for other/unknown subgroup analyses was only adjusted for age, oral contraceptives, *E. coli, Klebsiella spp*, and non-susceptibility to antibiotics due to small sample size and lack of convergence. | | | | | |
| ^3^At index date (date of the earliest record for the index uUTI). | | | | | |
| ^4^In year prior to index date (defined as the date of the earliest record for the index uUTI). | | | | | |
| ^5^For each antibiotic class, the comparison group did not receive an antibiotic of that class in the prior year. | | | | | |
| ^6^Other organisms included *Proteus mirabilis* (4.29%), *Citrobacter spp* (1.59%), *Enterobacter spp* (1.51*%), Enterococcus spp* (0.92%), *Pseudomonas aeroguinosa* (0.46%), Coagulase-negative *Staphylococcus spp* (0.32%). More than one organism was isolated from 1.83% of samples. | | | | | |
| ^7^*E. coli* isolated vs another organism isolated. | | | | | |
| ^8^*Klebsiella spp* isolated vs another organism isolated. | | | | | |
| ^9^Antibiotic classes were aminoglycosides, carbapenems, cephalosporins, fluroquinolones, fosfomycin, nitrofurantoin, penicillins, trimethoprim - sulfamethoxazole, others (aztreonam, colistin, daptomycin, linezolid, tigecycline, vancomycin). | | | | | |
